# Lower testosterone levels are associated with higher risk of death in men

**DOI:** 10.1093/emph/eoac044

**Published:** 2022-12-26

**Authors:** Michael P Muehlenbein, Jeffrey Gassen, Eric C Shattuck, Corey S Sparks

**Affiliations:** Department of Anthropology, Baylor University, TX, USA; Department of Anthropology, Baylor University, TX, USA; Institute for Health Disparities Research, University of Texas at San Antonio, TX, USA; Department of Public Health, University of Texas at San Antonio, TX, USA; Department of Demography, University of Texas at San Antonio, TX, USA

**Keywords:** testosterone, NHANES, mortality, chronic disease

## Abstract

**Background and Objectives:**

Testosterone plays an important role in regulating male development, reproduction and health. Declining levels across the lifespan may reflect, or even contribute to, chronic disease and mortality in men.

**Methodology:**

Relationships between testosterone levels and male mortality were analyzed using data from multiple samples of the cross-sectional National Health and Nutrition Examination Survey (*n* = 10 225). Target outcomes included known deaths from heart disease, malignant neoplasms, chronic lower respiratory diseases, cerebrovascular diseases, Alzheimer’s disease, diabetes mellitus, influenza and pneumonia, kidney diseases, and accidents or unintentional injuries.

**Results:**

Results of discrete-time hazard models revealed that lower levels of testosterone were related to higher mortality for the majority of disease categories in either an age-dependent or age-independent fashion. Analysis of all-cause mortality—which included deaths from any known disease—also revealed greater general risk for those with lower testosterone levels. For most disease categories, the hazard associated with low testosterone was especially evident at older ages when mortality from that particular ailment was already elevated. Notably, testosterone levels were not related to mortality risk for deaths unrelated to chronic disease (i.e. accidents and injuries).

**Conclusions and Implications:**

While the causal direction of relationships between testosterone and mortality risk remains unclear, these results may reflect the decline in testosterone that accompanies many disease states. Accordingly, the relationship between testosterone and male mortality may be indirect; ill individuals are expected to have both lower testosterone and higher mortality risk.

## Teaser

Testosterone plays a key role in regulating health and development, particularly for males. Numerous health problems, such as infections and chronic diseases—like diabetes and cancer— have been linked to decreases in testosterone levels. Several studies have also identified relationships between low testosterone levels and higher risk of death in men. While low testosterone could cause increases in men’s mortality risk, it is also possible that low testosterone levels may just be a sign of poor health. Men with illness or disease are expected to have both low testosterone levels and higher risk of death. The current research examined relationships between testosterone levels and risk of mortality from several causes of death in 10 255 men. Results revealed that for most mortality categories, including heart disease, cancer, cerebrovascular disease (such as strokes), influenza and pneumonia, and Alzheimer’s disease, lower testosterone levels were associated with higher risk of death, particularly among older men. That low testosterone is related to mortality risk from many unique diseases may indicate that low levels of this hormone reflect poor general health. However, additional research is needed to determine whether testosterone levels directly contribute to, or are just a sign of, disease and mortality risk in men.

## INTRODUCTION

Life-history theory provides a powerful framework for understanding how organisms invest energetic resources in completing multiple fitness-critical tasks, such as growth, reproduction and somatic maintenance across the lifespan [1, 2]. Because resources are finite, energy allocated to one domain cannot be used in another. Accordingly, organisms’ investment strategies often involve making trade-offs between life-history domains; optimal investment strategies differ by species [1], sex [3], age [4] and due to a variety of environmental and internal factors [5, 6]. For example, when adequate resources are available, higher payouts from investment in growth are expected during adolescence compared to later in life [7]. Further, in the context of infectious illness, increasing the energy allocated toward immune function and somatic repair is likely to yield greater fitness benefits than enhanced investment in reproduction [8, 9].

Testosterone is central to male mammalian life-history trajectories and trade-offs [9]. Specifically, testosterone plays an important role in facilitating many aspects of reproductive investment, such as the development of secondary sex characteristics [10], spermatogenesis [11] and intrasexual competition [12]. Research finds that elevated investment in these testosterone-mediated activities may, in turn, bear costs for somatic maintenance and survival [2, 9]. This trade-off is reflected in sex differences in immune function and mortality. Given that males of most mammalian species have a lower obligate energetic investment in offspring than females, they typically invest more heavily in mating effort [13]. Consistent with a trade-off between reproduction and somatic maintenance, males’ greater investment in mating is often accompanied by lower immunocompetence [14] and higher age-specific mortality relative to females [15].

Some theories and research suggest that variation in mating effort between males of the same species may also contribute to individual differences in health and immune function [16]. However, the extent to which testosterone mediates such trade-offs between mating and health in males has begun to come into question. For example, the immunocompetence handicap hypothesis proposes that testosterone possesses dual functions of both promoting elaborate sexually selected traits and suppressing immune function [17]. Therefore, only males of high genetic quality should be capable of withstanding the immunosuppressive effects of high testosterone levels to develop robust sexual signals. While this hypothesis has gained and maintained wide attention, support for some of its core tenets is surprisingly lacking [9, 14]. Most notably, testosterone has been found to be more immunomodulatory than chiefly immunosuppressive [18], and evidence that differences between males in levels of testosterone within the normal physiological range predict immune function is inconsistent [9], particularly in humans [19, 20]. (It should be considered that null findings of relationships between testosterone and immune function could be interpreted as supporting the immunocompetence handicap hypothesis. That is, if testosterone is truly immunosuppressive, it is possible that the suppressive effects of high testosterone decrease high-quality males’ immune responsiveness to a level similar to that of low-quality males. In other words, high-quality males would have more robust immune function were it not for the immunosuppressive effects of elevated testosterone.)

This previous work suggests that although testosterone is a key mediator of investment in males’ sexual development and mating behavior [10–12], it may not be directly responsible for decrements in immunity that occur in the context of elevated reproductive effort. Furthermore, research on testosterone-replacement therapy in men also seems to suggest that artificially raising testosterone levels does not consistently result in substantial changes to general health or mortality risk [21], which would be predicted by theories of testosterone-mediated trade-offs against somatic effort. Nonetheless, endogenous testosterone levels do appear to be linked to male health [22, 23], and changing levels during the lifespan could reflect alterations in trade-off investments with resultant altered physiological functioning that may contribute to mortality. Previous studies examining links between testosterone levels and men’s mortality risk have yielded mixed results: many find increased risk from certain causes at low testosterone levels, some find decreased risk, and still, others find no significant relationship between testosterone and mortality [23–25]. Heterogeneity in such research results is likely attributable to differences in sample characteristics (e.g. mean age), but may also reflect bidirectionality in relationships between androgens and health.

Testosterone levels decrease—often dramatically—in response to many infectious illnesses (e.g. COVID-19, HIV/AIDS) [22] and chronic diseases (e.g. cancer, diabetes mellitus) [26]. Even among otherwise healthy individuals, more minor health challenges—such as disrupted sleep [27], poor diet [28] or stress [29]—are capable of altering testosterone levels. Accordingly, relationships between testosterone levels and mortality may be incidental. That is, people with underlying illness or poor general health are expected to have both lower testosterone levels and experience a greater risk for mortality. It is, therefore, possible that links between testosterone levels and male mortality do not necessarily reflect testosterone-mediated trade-offs between reproductive effort and survival. Instead, poor health and disease states may demand an increase in somatic investment that comes at the cost of diminished investment in reproduction, of which decreases in testosterone levels provide a useful proxy.

Preliminary evidence for an incidental role of testosterone in men’s mortality risk could be provided by data demonstrating that low testosterone levels are predictive of age-specific mortality from a wide range of chronic diseases and infectious illnesses. In addition to supporting that low testosterone levels serve as a proxy for poor health, and thus mortality risk, such results would also provide evidence against the hypothesis that high endogenous testosterone levels in men produce trade-offs between reproductive and somatic effort that compromise health.

In the current research, we explored relationships between testosterone levels and mortality using data from males who participated in the National Health and Nutrition Examination Survey (*n* = 10 225). Included in the analyses were known deaths from heart disease, malignant neoplasms, chronic lower respiratory diseases, cerebrovascular diseases, Alzheimer’s disease, diabetes mellitus, influenza and pneumonia, kidney diseases, and accidents or unintentional injuries. Relationships between testosterone levels and all-cause mortality—which included deaths from any disease category, but excluded accidents or injuries—were also examined. We predicted that low testosterone levels would be associated with elevated mortality risk from a broad range of causes, consistent with the hypothesis that testosterone levels provide a proxy for men’s overall health.

## METHODS

Data used in the current research were extracted from public-use NHANES files. The NHANES is a nationally representative cross-sectional probability sample of the noninstitutionalized population of the USA collected by the National Center for Health Statistics (NCHS). Data used in the current research were limited to NHANES waves that included serum testosterone measurements, including the NHANES III (1984–94) and the continuous NHANES (1999–2004, 2011–16). These data were merged with the latest National Death Index (NDI) documents available, which provide death certificate information for NHANES participants who had died up to the year 2019 (https://www.cdc.gov/nchs/data-linkage/mortality.htm).

NHANES mortality data are available for participants 18 years of age and older. If an individual did not have linked mortality data, or was still alive at the 2019 follow-up, they were treated as censored in the present analysis. Age at death for those who died was calculated using their age at the time of the survey, and their reported age at death. For those who were censored, their age at death was considered censored, and was calculated as their age in the year 2019.

The predictor of interest in the current research was serum total testosterone levels. The methods used by the CDC for analyzing testosterone in NHANES serum samples can be found at https://wwwn.cdc.gov/nchs/nhanes/. To control for the effects of baseline mortality risk factors, the following covariates were included in the present analyses: (i) age, (ii) race/ethnicity, (iii) body mass index, and (iv) education as a proxy for socioeconomic status. Data for C-reactive protein (CRP) were also included as a biomarker of inflammation. However, CRP data were limited to the NHANES III, 1999–2000, 2001–2002 and 2003–2004 waves (*n* = 2561). Accordingly, CRP was controlled for only in follow-up models on this truncated dataset; results of these analyses are referenced in the main text and reported in full in Supplemental File 2 (Tables S3–S5).

Smoking status was not included as a covariate in the primary analyses due to large amounts of missing data (46.7%); follow-up analyses with reduced sample sizes examined whether the pattern or significance of results changed when controlling for smoking status. (These follow-up analyses with the truncated sample revealed that only the relationship between testosterone levels and mortality from malignant neoplasms became nonsignificant when controlling for smoking status.)

Detailed information about sample and survey collection can be found on the NHANES website (https://www.cdc.gov/nchs/nhanes/index.htm). Age, race/ethnicity and education were each measured at the initial household interviews. Data for the remaining variables were subsequently collected during an appointment at a mobile examination clinic. We were unable to find specific information about the amount of time that passed between initial interviews and clinical appointments, but each occurred within the 2-year cycle of each wave. Race/ethnicity was coded as three dummy variables representing non-Hispanic White (reference), non-Hispanic Black, non-Hispanic other or multiracial, or Hispanic/Latino. Education was treated as a continuous variable representing the highest grade completed (1 = less than high school, 2 = high school and 3 = greater than high school).

## DATA ANALYSIS

R code for all analyses is included in Supplemental File 1. Discrete-time hazard modeling was employed involving a binomial regression model with a complementary log-log link function. This model measured the effects of the covariates on the hazard (*h*(*t*)) of dying during the period as a linear function of the covariates (*x*). The model was specified as


$$\begin{array}{l} log\left[-log\left(1-h(t)\right)\right]=x^{\prime }\beta \end{array}$$

where the *β* parameters measure the effect of each covariate on the hazard. The complementary log-log model has the advantage over the traditional logistic link function in that its effects are interpretable as hazard ratios, instead of odds ratios.

Data were first transformed into a person–period format with individuals contributing one period of risk for each 10-year segment of age they are exposed to the risk of death. Because the NHANES is a complex, multistage probability sample, Taylor series linearization methods were used to estimate all standard errors for model parameters [30]. NCHS provides sample stratum, primary sampling unit identifiers and sampling weights for each respondent. All models are estimated using survey analysis procedures in the *survey* package (v4.1-1) in the R statistical programming language [31]. Given the nonlinear change of hazard over age, a cubic B-spline basis was included using R package *splines* (v4.0.4).

## RESULTS

**Figure 1.**
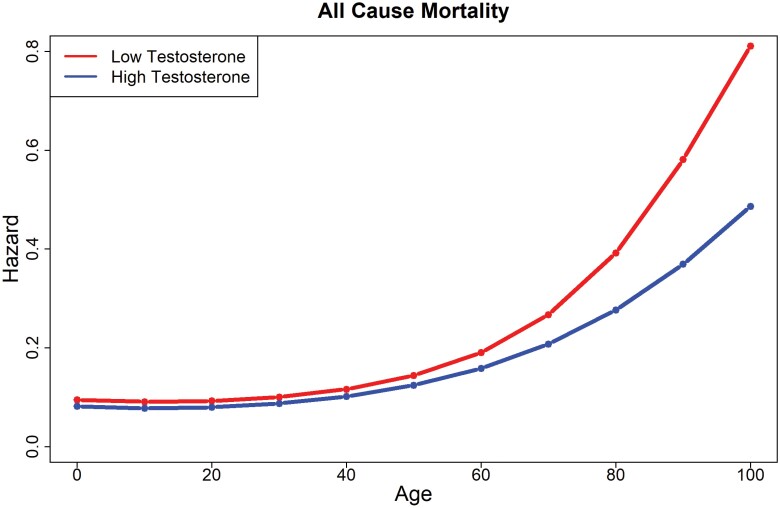
Hazard ratios by testosterone level for all-cause mortality.

**Figure 2.**
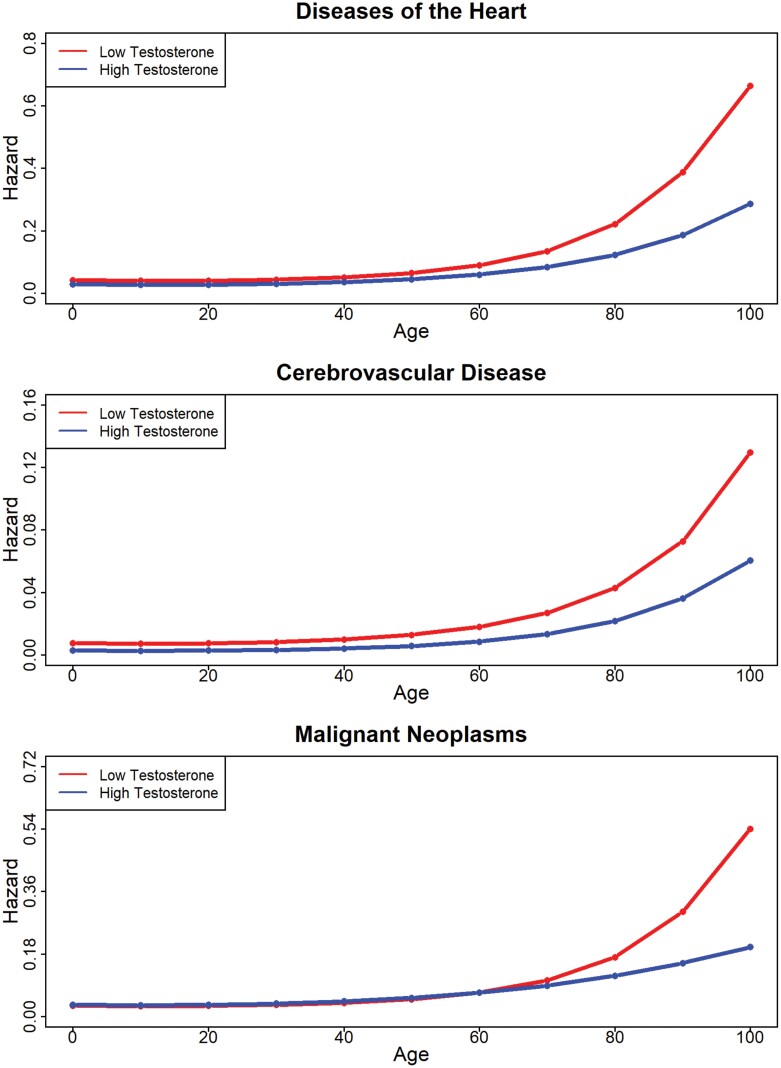
Hazard ratios by testosterone level for heart diseases, cerebrovascular diseases and malignant neoplasms.

**Figure 3.**
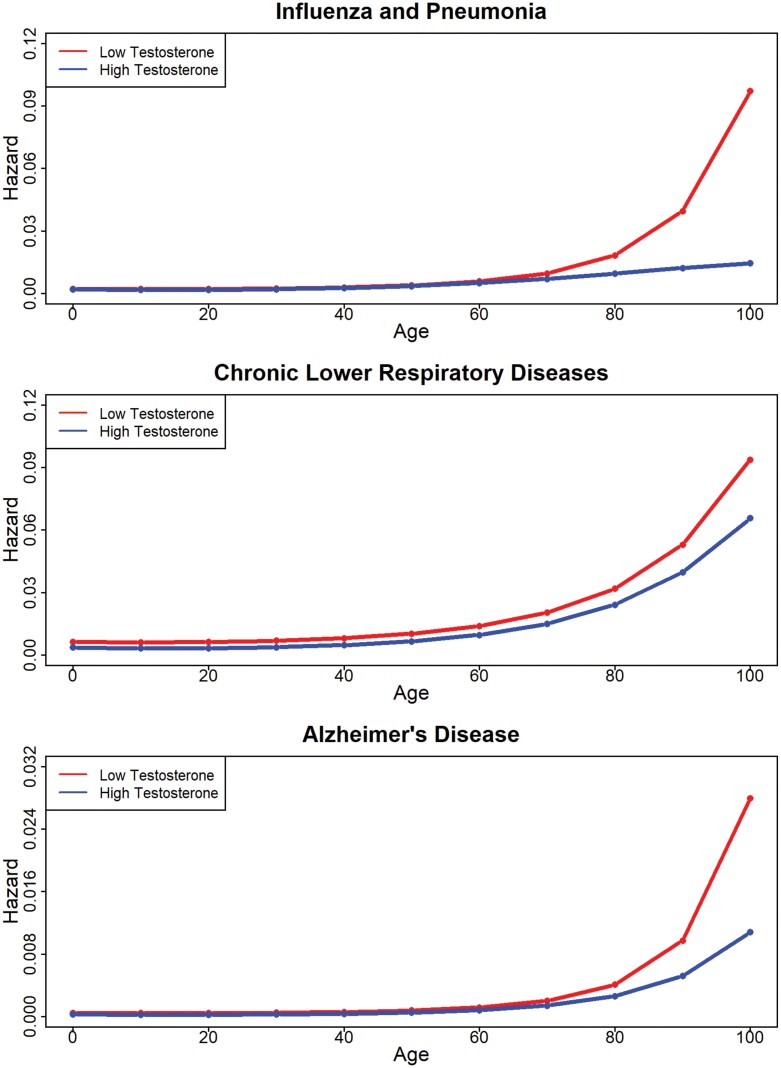
Hazard ratios by testosterone level for influenza/pneumonia, chronic lower respiratory diseases and Alzheimer’s disease.

Descriptive statistics are available in Tables S1–S2 in Supplemental File 2. Results of discrete-time hazard models adjusted for covariates are shown in Tables 1–3 and Figs. 1–3; these results are presented in text in the order of their appearance in tables and figures. For all-cause mortality (excluding deaths unrelated to disease), the main effect of testosterone was statistically significant (HR = 0.94 [0.88, 0.99]), but was qualified by an interaction between testosterone levels and the third-age spline (HR = 0.73 [0.63, 0.85]). As shown in Fig. 1, lower levels of testosterone were related to greater hazard, an effect that grew increasingly wide after age 60. The pattern and significance of these results did not change in the follow-up analyses of the truncated dataset that included CRP as a covariate (Table S2).

**Table 1.** Results of discrete-time hazard models for all-cause mortality, heart diseases, cardiovascular diseases and malignant neoplasms

	All-cause mortality (*n* = 1162)				Heart disease (*n* = 475)				Cerebrovascular disease (*n* = 86)				Malignant neoplasms (*n* = 395)			
Predictor	*β*	SE	*p*	Hazard	*β*	SE	*p*	Hazard	*β*	SE	*p*	Hazard	*β*	SE	*p*	Hazard
				Ratio (95% CIs)				Ratio (95% CIs)				Ratio (95% CIs)				Ratio (95% CIs)
Age Spline-1	−0.16	0.15	0.28	0.85 (0.63, 1.14)	−0.05	0.20	0.82	0.96 (0.64, 1.42)	−0.04	0.31	0.89	0.96 (0.52, 1.75)	−0.05	0.19	0.80	0.95 (0.66, 1.38)
Age Spline-2	0.10	0.34	0.76	1.11 (0.57, 2.14)	−0.11	0.44	0.79	0.89 (0.38, 2.10)	0.12	0.67	0.86	1.13 (0.30, 4.22)	−0.22	0.37	0.56	0.80 (0.39, 1.66)
Age Spline-3	3.77	0.37	<0.001***	43.31 (20.97, 89.44)	4.25	0.52	<0.001***	70.17 (25.36, 194.20)	2.73	0.99	0.01**	15.35 (2.22, 106.03)	4.88	0.52	<0.001***	131.97 (47.23, 368.80)
Testosterone	−0.06	0.03	0.048*	0.94 (0.88, 0.99)	−0.15	0.05	0.01**	0.86 (0.78, 0.96)	−0.38	0.12	0.002**	0.68 (0.54, 0.86)	0.04	0.03	0.12	1.04 (0.99, 1.10)
African American	−0.41	0.14	0.004**	0.67 (0.51, 0.87)	−0.26	0.16	0.11	0.77 (0.57, 1.06)	0.03	0.40	0.95	1.03 (0.47, 2.25)	−0.64	0.25	0.01*	0.53 (0.32, 0.87)
Other Ethnicity	−0.81	0.22	<0.001***	0.45 (0.29, 0.69)	−0.99	0.40	0.02*	0.37 (0.17, 0.81)	−0.73	0.69	0.30	0.48 (0.12, 1.87)	−0.83	0.43	0.06†	0.44 (0.19, 1.01)
Hispanic/Latino	−0.03	0.13	0.80	0.97 (0.75, 1.25)	0.06	0.22	0.78	1.06 (0.69, 1.64)	0.01	0.44	0.98	1.01 (0.43, 2.37)	-0.09	0.20	0.65	0.91 (0.61, 1.36)
Education	−0.43	0.07	<0.001***	0.65 (0.56, 0.75)	−0.51	0.12	<0.001***	0.60 (0.47, 0.76)	−0.38	0.18	0.04*	0.68 (0.48, 0.97)	−0.39	0.09	<0.001***	0.68 (0.57, 0.81)
Body Mass Index	−0.02	0.01	0.047*	0.98 (0.96, 0.99)	−0.01	0.02	0.61	0.99 (0.96, 1.02)	−0.13	0.05	0.02*	0.88 (0.80, 0.97)	−0.04	0.02	0.03*	0.96 (0.93, 1.00)
Age 1*Testosterone	−0.03	0.03	0.39	0.97 (0.91, 1.04)	−0.06	0.05	0.22	0.94 (0.86, 1.03)	−0.10	0.07	0.19	0.91 (0.79, 1.05)	−0.05	0.04	0.21	0.96 (0.89, 1.03)
Age 2*Testosterone	0.13	0.07	0.07	1.14 (0.99, 1.31)	0.15	0.10	0.14	1.16 (0.95, 1.42)	0.21	0.14	0.13	1.23 (0.94, 1.62)	0.21	0.07	0.003**	1.23 (1.08, 1.40)
Age 3*Testosterone	−0.31	0.08	<0.001***	0.73 (0.63, 0.85)	−0.34	0.12	0.01**	0.71 (0.57, 0.90)	0.05	0.17	0.77	1.05 (0.76, 1.46)	−0.55	0.11	<0.001***	0.57 (0.46, 0.72)

*Note:* Lower testosterone levels are associated with higher risk of death in men.

*p < 0.05, **p < 0.01, ***p < 0.001, †*p* < 0.06.

**Table 2.** Results of discrete-time hazard models for influenza and pneumonia, chronic lower respiratory diseases and Alzheimer’s disease

	Influenza and pneumonia (*n* = 29)				Chronic lower respiratory disease (*n* = 66)				Alzheimer’s disease (*n* = 38)			
Predictor	*β*	SE	p	Hazard	β	SE<	p	Hazard	β	SE	p	Hazard
				ratio (95% CIs)				ratio (95% CIs)				ratio (95% CIs)
Age Spline-1	0.20	0.39	0.60	1.22 (0.57, 2.63)	0.15	0.46	0.74	1.17 (0.47, 2.89)	0.49	0.26	0.07	1.63 (0.97, 2.71)
Age Spline-2	−1.04	0.69	0.13	0.35 (0.09, 1.35)	−0.05	1.20	0.96	0.95 (0.09, 9.88)	−0.95	0.61	0.12	0.39 (0.12, 1.27)
Age Spline-3	6.02	0.93	<0.001***	410.76 (66.20, 2548.82)	2.50	1.75	0.16	12.19 (0.40, 374.64)	4.64	0.89	<0.001***	103.60 (17.94, 598.22)
Testosterone	−0.06	0.10	0.54	0.94 (0.77, 1.15)	−0.23	0.13	0.09	0.80 (0.61, 1.03)	−0.21	0.11	0.05†	0.81 (0.66, 1.00)
African American	−0.02	0.51	0.97	0.98 (0.36, 2.65)	−1.49	0.51	0.005**	0.22 (0.08, 0.61)	−1.76	0.64	0.01**	0.17 (0.05, 0.60)
Other Ethnicity	0.27	0.89	0.76	1.31 (0.23, 7.46)	0.08	0.56	0.88	1.09 (0.37, 3.24)	−15.43	0.20	<0.001***	0.00 (0.00, 0.00)
Hispanic/Latino	0.29	0.68	0.67	1.33 (0.35, 5.05)	0.23	0.42	0.59	1.25 (0.56, 2.83)	−0.40	0.57	0.48	0.67 (0.22, 2.05)
Education	−0.58	0.25	0.02*	0.56 (0.34, 0.92)	−0.61	0.18	<0.001***	0.54 (0.38, 0.77)	−0.25	0.23	0.29	0.78 (0.49, 1.23)
Body Mass Index	0.01	0.04	0.84	1.01 (0.94, 1.08)	0.03	0.05	0.49	1.03 (0.94, 1.14)	−0.12	0.04	0.01**	0.89 (0.82, 0.97)
Age 1*Testosterone	−0.16	0.08	0.06†	0.85 (0.73, 1.00)	−0.14	0.08	0.09	0.87 (0.75, 1.02)	−0.22	0.05	<0.001***	0.81 (0.73, 0.89)
Age 2*Testosterone	0.49	0.16	0.004**	1.62 (1.18, 2.24)	0.23	0.21	0.29	1.26 (0.83, 1.91)	0.38	0.12	0.002**	1.46 (1.16, 1.85)
Age 3*Testosterone	−0.74	0.25	0.005**	0.48 (0.29, 0.78)	0.08	0.34	0.82	1.08 (0.55, 2.11)	−0.18	0.17	0.27	0.83 (0.60, 1.15)

*Note.* Lower testosterone levels are associated with higher risk of death in men.

*p < 0.05, **p < 0.01, ***p < 0.001, †*p* < 0.06.

**Table 3.** Results of discrete-time hazard models for diabetes mellitus, kidney disease and accidents/injuries

	Diabetes (*n* = 49)				Kidney disease (*n* = 24)				Accidents/injuries (*n* = 52)			
Predictor	*β*	SE	*p*	Hazard	*β*	SE	*p*	Hazard	*β*	SE	*p*	Hazard
				ratio (95% CIs)				ratio (95% CIs)				ratio (95% CIs)
Age Spline-1	−0.52	0.74	0.48	0.59 (0.14, 2.50)	−0.01	0.38	0.97	0.99 (0.47, 2.09)	0.37	0.50	0.47	1.44 (0.54, 3.86)
Age Spline-2	1.41	1.88	0.46	4.10 (0.10, 162.89)	0.01	1.11	0.99	1.01 (0.11, 8.98)	−1.16	1.52	0.45	0.31 (0.02, 6.19)
Age Spline-3	1.16	2.25	0.61	3.18 (0.04, 263.91)	3.68	2.11	0.09	39.58 (0.63, 2481.40)	4.81	1.41	0.001**	122.93 (7.80, 1937.20)
Testosterone	−0.14	0.18	0.44	0.87 (0.61, 1.24)	0.03	0.14	0.80	1.04 (0.79, 1.36)	0.05	0.04	0.22	1.06 (0.97, 1.15)
African American	−0.33	0.50	0.51	0.72 (0.27, 1.93)	0.52	0.60	0.39	1.67 (0.51, 5.45)	−0.44	0.54	0.42	0.65 (0.22, 1.87)
Other Ethnicity	−15.42	0.32	<0.001***	0.00 (0.00, 0.00)	−0.06	1.22	0.96	0.94 (0.09, 10.33)	−2.45	0.75	0.002**	0.09 (0.02, 0.37)
Hispanic/Latino	−0.43	0.48	0.38	0.65 (0.25, 1.68)	−0.60	0.63	0.34	0.55 (0.16, 1.89)	−0.09	0.59	0.88	0.92 (0.29, 2.89)
Education	−0.40	0.23	0.09	0.67 (0.43, 1.06)	−0.64	0.30	0.04*	0.53 (0.29, 0.95)	−0.31	0.30	0.30	0.73 (0.40, 1.32)
Body Mass Index	−0.01	0.04	0.75	0.99 (0.90, 1.07)	0.03	0.04	0.51	1.03 (0.95, 1.11)	0.00	0.04	0.97	1.00 (0.93, 1.07)
Age 1*Testosterone	0.02	0.19	0.92	1.02 (0.70, 1.47)	−0.06	0.06	0.30	0.94 (0.84, 1.06)	−0.03	0.10	0.78	0.97 (0.80, 1.19)
Age 2*Testosterone	−0.07	0.44	0.87	0.93 (0.39, 2.20)	0.13	0.20	0.51	1.14 (0.77, 1.68)	0.08	0.31	0.79	1.09 (0.59, 2.00)
Age 3*Testosterone	0.05	0.36	0.89	1.05 (0.52, 2.11)	−0.15	0.44	0.74	0.86 (0.36, 2.06)	−0.50	0.31	0.11	0.61 (0.33, 1.11)

*Note*: Lower testosterone levels are associated with higher risk of death in men.

*p < 0.05, **p < 0.01, ***p < 0.001, †*p* < 0.06.

A similar pattern of results was found for mortality from heart disease (Fig. 2); the main effect of testosterone was significant (HR = 0.86 [0.78, 0.96]), as was the interaction with the third-age spline (HR = 0.71 [0.57, 0.90]). Again, the greater mortality risk among men with lower testosterone levels was especially pronounced after age 60.

For mortality from cerebrovascular diseases, lower levels of testosterone were, again, associated with higher hazard (main effect: HR = 0.68 [0.54, 0.86]). Although no interactions between testosterone levels and age reached statistical significance, the pattern of age-dependent changes in the hazard associated with low testosterone levels resembled that of heart disease and all-cause mortality (Fig. 2). Results for deaths from malignant neoplasms revealed a nonsignificant main effect of testosterone (HR = 1.04 [0.99, 1.10]), but significant interactions between testosterone levels and the second- and third-age splines (second: HR = 1.23 [1.08, 1.40]; third: HR = 0.57 [0.46, 0.72]). As depicted in Fig. 2, lower testosterone levels were associated with a greater proportional hazard only at older ages (i.e. age of >80). Follow-up analyses of the truncated dataset that included CRP-supported results for these disease categories (Table S3).

For mortality from influenza and pneumonia, the main effect of testosterone was not statistically significant (HR = 0.94 [0.77, 1.15]), but there were significant interactions between testosterone and the final two age splines (spline-2: HR = 1.62 [1.18, 2.24]; spline-3: HR = 0.48 [0.29, 0.78]). Specifically, lower testosterone levels were associated with greater predicted hazard beginning at age 80, an effect that grew wider thereafter (Fig. 3). While testosterone was not related to mortality from chronic lower respiratory disease in the primary analysis, results of the follow-up analysis controlling for CRP revealed greater hazard among men with lower levels of testosterone, especially after age of 60 (spline-1: HR = 0.78 [0.65, 0.93]; spline-2: HR = 1.70 [1.12, 2.57]; Table S4). For deaths from Alzheimer’s disease, there were significant interactions between testosterone levels and the first two age splines (spline one: HR = 0.81 [0.73, 0.89]; spline two: HR = 1.46 [1.16, 1.85]). As is shown in Fig. 3, lower testosterone levels predicted greater hazard from this disease category only after age 80. Aside from the results for chronic lower respiratory disease, controlling for CRP in the truncated dataset did not otherwise change the pattern or significance of these results (Table S4).

No significant effects of testosterone levels were found for the risk of mortality from diabetes mellitus or kidney diseases (see Table S5 and Fig. S1 in Supplemental File 2). This was also true for the follow-up analyses controlling for CRP (Table S5). Testosterone levels were not related to hazards from accidents or unintentional injuries in either the primary or follow-up analyses, perhaps indicating that the relationship between testosterone and mortality is specific to deaths from chronic and infectious diseases.

## DISCUSSION

The current research builds on previous work that has explored relationships between testosterone levels and mortality in men by examining the effects of testosterone on multiple mortality categories, including heart disease, malignant neoplasms, chronic lower respiratory diseases, cerebrovascular diseases, Alzheimer’s disease, diabetes mellitus, influenza and pneumonia, kidney diseases, and accidents or unintentional injuries [24]. The present research also builds on previous studies by analyzing links between testosterone levels and male mortality in a large, nationally representative US sample of NHANES participants. While prior work using NHANES data has examined relationships between men’s testosterone levels and specific mortality categories (e.g. cardiovascular disease) in a limited subset of NHANES waves and/or a specific sub-sample of participants (e.g. men with metabolic disorders) [32–38], to our knowledge, the current analyses are the first to include data from all waves for which serum testosterone levels were assayed (1984–94, 1999–2004, 2011–16), all eligible participants with linked mortality data (*n* = 10 225), and most importantly, all publicly available mortality categories. Moreover, the current research is also the first to analyze relationships between NHANES participants’ testosterone levels and mortality using the latest NDI mortality data (i.e. 2019 update).

Results revealed that for all-cause mortality (i.e. including all deaths related to disease), as well as for deaths specifically from heart disease, malignant neoplasms, cerebrovascular diseases, influenza/pneumonia, and Alzheimer’s disease, lower testosterone levels were associated with elevated mortality risk. While relationships between testosterone levels and mortality from chronic lower respiratory disease did not reach statistical significance in the primary analyses, a follow-up analysis on a subset of waves for which CRP data were available to include as a covariate revealed greater hazard among men with lower testosterone levels. The pattern and significance of results for other disease categories did not change when controlling for CRP levels. For nearly all disease categories (with the exception of cerebrovascular disease), the hazard associated with lower testosterone levels was most prominent at older ages. Specifically, the hazard associated with lower testosterone levels tended to increase sharply either after age 60 (all-cause, heart disease) or 80 (cancer, influenza and pneumonia, Alzheimer’s disease).

Among the disease categories, significant relationships were not found between testosterone levels and risk of mortality from diabetes mellitus or kidney diseases, either in the full sample or sub-sample with CRP included as a covariate. Testosterone levels also did not predict hazards from accidents and unintentional injuries, the sole cause of mortality unrelated to disease included in the dataset. In addition to unintentional injuries, this mortality category included deaths attributable to motor vehicle and other transport accidents, falls, drowning, accidental firearm discharges, and exposure to fire and noxious substances. Although only speculative, it is possible that these results highlight a boundary condition in the relationship between testosterone and mortality. That is, the link between testosterone levels and mortality risk may be specific to deaths from disease. Conversely, it is also possible that the total effect of testosterone on accidents and injuries is masked in the present analyses. Men experience higher rates of accidental deaths than women [39], which is likely driven, in part, by testosterone’s effects on risk-taking behavior [40]. In other words, testosterone may play a role in promoting sex differences in accidental deaths, but no differences between men.

Given that the NHANES data are cross-sectional, causal claims cannot be made about the direction of relationships found between testosterone and mortality. However, relationships between testosterone and mortality were found across nearly all disease categories seem to suggest that low levels of testosterone may serve as a proxy for poor overall health. This possibility is consistent with research finding that testosterone levels decline in response to numerous disease states. Ill individuals are expected to have both higher mortality and lower levels of testosterone. Further research is needed to determine if and when low testosterone levels are a cause or consequence of health problems that elevate one’s mortality risk.

Confirmation that low testosterone is more of a symptom than a cause of chronic health problems during aging would challenge the marketing of testosterone supplementation as a panacea for men’s health issues, ranging from low sexual function, to unideal body composition, to general aging [41]. Such findings would also raise questions about the utility of testosterone-replacement therapy as a means of reducing mortality risk in aged men. Studies on the impact of testosterone-replacement therapy on male mortality, to date, have yielded mixed results [23–25]. For example, while some previous research has suggested that testosterone-replacement therapy increases the risk of cardiovascular disease, many of these studies were underpowered or not specifically designed to address this question [42]. Separate studies have found no impact of testosterone supplementation on cardiovascular disease [21], or even a protective effect for hypogonadal men [43].

The present research has limitations that should be considered. First, because the NDI’s public-use mortality files only provide information about broad disease categories (e.g. heart disease, cancer), nuanced relationships between testosterone levels and more specific causes of death within each category could not be explored. For example, the category of mortality from malignant neoplasms used in the current study includes deaths from prostate cancer, and androgens play a role in the etiology of this disease [44]. Accordingly, it is possible that if examined separately, relationships between testosterone levels and prostate cancer would follow the opposite pattern as that observed here. However, it should be noted that recent systematic reviews and meta-analyses do suggest that neither high endogenous testosterone levels [45], nor testosterone supplementation [46, 47], are predictive of prostate cancer development. Another limitation of this research is that only a single measure of testosterone was collected, which may introduce sources of bias or error as testosterone levels vary diurnally [48], seasonally [49] and in response to myriad life events [27, 29]. Further, because it is cross-sectional, the current study cannot lend insights into whether the organizational effects of testosterone influence mortality risk later in life. Some research finds that early-life castration extends men’s overall lifespans [50], which at least suggests that the organizational effects of testosterone, whether biological or behavioral, play a role in promoting aging.

In sum, the results of the current study found that low testosterone levels in men were associated with an elevated risk of mortality from a diverse range of diseases, particularly later in life. Additional research is needed to confirm the current findings in a wider body of publicly available datasets that offer larger sample sizes and information about additional causes of mortality. Nonetheless, the present results provide support for a link between testosterone and male mortality in a large nationally representative sample. This research may lay the groundwork for future studies to further untangle intricate relationships between sex hormones and health across the lifespan.

## Supplementary Material

eoac044_suppl_Supplementary_File_S1

eoac044_suppl_Supplementary_File_S2

## Data Availability

Data are publicly available and R code is submitted as a supplemental file.
