# Supplementary material for: Lower testosterone levels are associated with higher risk of death in men
Source: Evol Med Public Health. 2022 Dec 26;11(1):30–41. doi: 10.1093/emph/eoac044 (PMC9938530; doi:10.1093/emph/eoac044)
Supplement: eoac044_suppl_Supplementary_File_S1 [file eoac044_suppl_supplementary_file_s1.docx]

**Supplemental information for:**

Lower testosterone levels are associated with higher risk of death in men

**READ-IN PROGRAM FOR 2019 MORTALITY FILES**

#install.packages("readr") #if package is not installed already, then uncomment and run this line

#install.packages("dplyr") #if package is not installed already, then uncomment and run this line

library(readr)

library(dplyr)

# the location where the .DAT file is saved:

setwd("C:/Users/Xaust/Box/NHANES/Updated Mortality Files 2015")

# remove all objects from the R environment

rm(list=ls())

################

#NHANES III#

################

srvyin <- paste("NHANES_III_MORT_2019_PUBLIC.dat") # full .DAT name here

srvyout <- "nh3mort19" # shorthand dataset name here

# read in the fixed-width format ASCII file

dsn <- read_fwf(file=srvyin,

col_types = "ciiiiiiiddii",

fwf_cols(publicid = c(1,14),

eligstat = c(15,15),

mortstat = c(16,16),

ucod_leading = c(17,19),

diabetes = c(20,20),

hyperten = c(21,21),

dodqtr = c(22,22),

dodyear = c(23,26),

wgt_new = c(27,34),

sa_wgt_new = c(35,42),

permth_int = c(43,45),

permth_exm = c(46,48)

),

na = "."

)

# create the ID (SEQN) for the NHANES surveys

dsn$seqn <- substr(dsn$publicid,1,5)

# NOTE: SEQN is the unique ID for NHANES.

#Drop NHIS variables

dsn <- select(dsn, -publicid)

dsn <- select(dsn, -dodqtr)

dsn <- select(dsn, -dodyear)

dsn <- select(dsn, -wgt_new)

dsn <- select(dsn, -sa_wgt_new)

# Structure and contents of data

str(dsn)

# Variable frequencies

#ELIGSTAT: Eligibility Status for Mortality Follow-up

table(dsn$eligstat)

#1 = "Eligible"

#2 = "Under age 18, not available for public release"

#3 = "Ineligible"

#MORTSTAT: Final Mortality Status

table(dsn$mortstat, useNA="ifany")

# 0 = Assumed alive

# 1 = Assumed deceased

# <NA> = Ineligible or under age 18

#UCOD_LEADING: Underlying Cause of Death: Recode

table(dsn$ucod_leading, useNA="ifany")

# 1 = Diseases of heart (I00-I09, I11, I13, I20-I51)

# 2 = Malignant neoplasms (C00-C97)

# 3 = Chronic lower respiratory diseases (J40-J47)

# 4 = Accidents (unintentional injuries) (V01-X59, Y85-Y86)

# 5 = Cerebrovascular diseases (I60-I69)

# 6 = Alzheimer's disease (G30)

# 7 = Diabetes mellitus (E10-E14)

# 8 = Influenza and pneumonia (J09-J18)

# 9 = Nephritis, nephrotic syndrome and nephrosis (N00-N07, N17-N19, N25-N27)

# 10 = All other causes (residual)

# <NA> = Ineligible, under age 18, assumed alive, or no cause of death data

#DIABETES: Diabetes Flag from Multiple Cause of Death (MCOD)

table(dsn$diabetes, useNA="ifany")

# 0 = No - Condition not listed as a multiple cause of death

# 1 = Yes - Condition listed as a multiple cause of death

# <NA> = Assumed alive, under age 18, ineligible for mortality follow-up, or MCOD not available

#HYPERTEN: Hypertension Flag from Multiple Cause of Death (MCOD)

table(dsn$hyperten, useNA="ifany")

# 0 = No - Condition not listed as a multiple cause of death

# 1 = Yes - Condition listed as a multiple cause of death

# <NA> = Assumed alive, under age 18, ineligible for mortality follow-up, or MCOD not available

# Re-name the dataset, DSN, to the short survey name then remove other R objects

assign(paste0(srvyout), dsn)

rm(dsn, srvyin, srvyout)

################

#1999#

################

srvyin <- paste("NHANES_1999_2000_MORT_2019_PUBLIC.dat") # full .DAT name here

srvyout <- "nh99mort19" # shorthand dataset name here

# read in the fixed-width format ASCII file

dsn <- read_fwf(file=srvyin,

col_types = "ciiiiiiiddii",

fwf_cols(publicid = c(1,14),

eligstat = c(15,15),

mortstat = c(16,16),

ucod_leading = c(17,19),

diabetes = c(20,20),

hyperten = c(21,21),

dodqtr = c(22,22),

dodyear = c(23,26),

wgt_new = c(27,34),

sa_wgt_new = c(35,42),

permth_int = c(43,45),

permth_exm = c(46,48)

),

na = "."

)

# create the ID (SEQN) for the NHANES surveys

dsn$seqn <- substr(dsn$publicid,1,5)

# NOTE: SEQN is the unique ID for NHANES.

#Drop NHIS variables

dsn <- select(dsn, -publicid)

dsn <- select(dsn, -dodqtr)

dsn <- select(dsn, -dodyear)

dsn <- select(dsn, -wgt_new)

dsn <- select(dsn, -sa_wgt_new)

# Structure and contents of data

str(dsn)

# Variable frequencies

#ELIGSTAT: Eligibility Status for Mortality Follow-up

table(dsn$eligstat)

#1 = "Eligible"

#2 = "Under age 18, not available for public release"

#3 = "Ineligible"

#MORTSTAT: Final Mortality Status

table(dsn$mortstat, useNA="ifany")

# 0 = Assumed alive

# 1 = Assumed deceased

# <NA> = Ineligible or under age 18

#UCOD_LEADING: Underlying Cause of Death: Recode

table(dsn$ucod_leading, useNA="ifany")

# 1 = Diseases of heart (I00-I09, I11, I13, I20-I51)

# 2 = Malignant neoplasms (C00-C97)

# 3 = Chronic lower respiratory diseases (J40-J47)

# 4 = Accidents (unintentional injuries) (V01-X59, Y85-Y86)

# 5 = Cerebrovascular diseases (I60-I69)

# 6 = Alzheimer's disease (G30)

# 7 = Diabetes mellitus (E10-E14)

# 8 = Influenza and pneumonia (J09-J18)

# 9 = Nephritis, nephrotic syndrome and nephrosis (N00-N07, N17-N19, N25-N27)

# 10 = All other causes (residual)

# <NA> = Ineligible, under age 18, assumed alive, or no cause of death data

#DIABETES: Diabetes Flag from Multiple Cause of Death (MCOD)

table(dsn$diabetes, useNA="ifany")

# 0 = No - Condition not listed as a multiple cause of death

# 1 = Yes - Condition listed as a multiple cause of death

# <NA> = Assumed alive, under age 18, ineligible for mortality follow-up, or MCOD not available

#HYPERTEN: Hypertension Flag from Multiple Cause of Death (MCOD)

table(dsn$hyperten, useNA="ifany")

# 0 = No - Condition not listed as a multiple cause of death

# 1 = Yes - Condition listed as a multiple cause of death

# <NA> = Assumed alive, under age 18, ineligible for mortality follow-up, or MCOD not available

# Re-name the dataset, DSN, to the short survey name then remove other R objects

assign(paste0(srvyout), dsn)

rm(dsn, srvyin, srvyout)

################

#2001#

################

srvyin <- paste("NHANES_2001_2002_MORT_2019_PUBLIC.dat") # full .DAT name here

srvyout <- "nh01mort19" # shorthand dataset name here

# read in the fixed-width format ASCII file

dsn <- read_fwf(file=srvyin,

col_types = "ciiiiiiiddii",

fwf_cols(publicid = c(1,14),

eligstat = c(15,15),

mortstat = c(16,16),

ucod_leading = c(17,19),

diabetes = c(20,20),

hyperten = c(21,21),

dodqtr = c(22,22),

dodyear = c(23,26),

wgt_new = c(27,34),

sa_wgt_new = c(35,42),

permth_int = c(43,45),

permth_exm = c(46,48)

),

na = "."

)

# create the ID (SEQN) for the NHANES surveys

dsn$seqn <- substr(dsn$publicid,1,5)

# NOTE: SEQN is the unique ID for NHANES.

#Drop NHIS variables

dsn <- select(dsn, -publicid)

dsn <- select(dsn, -dodqtr)

dsn <- select(dsn, -dodyear)

dsn <- select(dsn, -wgt_new)

dsn <- select(dsn, -sa_wgt_new)

# Structure and contents of data

str(dsn)

# Variable frequencies

#ELIGSTAT: Eligibility Status for Mortality Follow-up

table(dsn$eligstat)

#1 = "Eligible"

#2 = "Under age 18, not available for public release"

#3 = "Ineligible"

#MORTSTAT: Final Mortality Status

table(dsn$mortstat, useNA="ifany")

# 0 = Assumed alive

# 1 = Assumed deceased

# <NA> = Ineligible or under age 18

#UCOD_LEADING: Underlying Cause of Death: Recode

table(dsn$ucod_leading, useNA="ifany")

# 1 = Diseases of heart (I00-I09, I11, I13, I20-I51)

# 2 = Malignant neoplasms (C00-C97)

# 3 = Chronic lower respiratory diseases (J40-J47)

# 4 = Accidents (unintentional injuries) (V01-X59, Y85-Y86)

# 5 = Cerebrovascular diseases (I60-I69)

# 6 = Alzheimer's disease (G30)

# 7 = Diabetes mellitus (E10-E14)

# 8 = Influenza and pneumonia (J09-J18)

# 9 = Nephritis, nephrotic syndrome and nephrosis (N00-N07, N17-N19, N25-N27)

# 10 = All other causes (residual)

# <NA> = Ineligible, under age 18, assumed alive, or no cause of death data

#DIABETES: Diabetes Flag from Multiple Cause of Death (MCOD)

table(dsn$diabetes, useNA="ifany")

# 0 = No - Condition not listed as a multiple cause of death

# 1 = Yes - Condition listed as a multiple cause of death

# <NA> = Assumed alive, under age 18, ineligible for mortality follow-up, or MCOD not available

#HYPERTEN: Hypertension Flag from Multiple Cause of Death (MCOD)

table(dsn$hyperten, useNA="ifany")

# 0 = No - Condition not listed as a multiple cause of death

# 1 = Yes - Condition listed as a multiple cause of death

# <NA> = Assumed alive, under age 18, ineligible for mortality follow-up, or MCOD not available

# Re-name the dataset, DSN, to the short survey name then remove other R objects

assign(paste0(srvyout), dsn)

rm(dsn, srvyin, srvyout)

################

#2003#

################

srvyin <- paste("NHANES_2003_2004_MORT_2019_PUBLIC.dat") # full .DAT name here

srvyout <- "nh03mort19" # shorthand dataset name here

# read in the fixed-width format ASCII file

dsn <- read_fwf(file=srvyin,

col_types = "ciiiiiiiddii",

fwf_cols(publicid = c(1,14),

eligstat = c(15,15),

mortstat = c(16,16),

ucod_leading = c(17,19),

diabetes = c(20,20),

hyperten = c(21,21),

dodqtr = c(22,22),

dodyear = c(23,26),

wgt_new = c(27,34),

sa_wgt_new = c(35,42),

permth_int = c(43,45),

permth_exm = c(46,48)

),

na = "."

)

# create the ID (SEQN) for the NHANES surveys

dsn$seqn <- substr(dsn$publicid,1,5)

# NOTE: SEQN is the unique ID for NHANES.

#Drop NHIS variables

dsn <- select(dsn, -publicid)

dsn <- select(dsn, -dodqtr)

dsn <- select(dsn, -dodyear)

dsn <- select(dsn, -wgt_new)

dsn <- select(dsn, -sa_wgt_new)

# Structure and contents of data

str(dsn)

# Variable frequencies

#ELIGSTAT: Eligibility Status for Mortality Follow-up

table(dsn$eligstat)

#1 = "Eligible"

#2 = "Under age 18, not available for public release"

#3 = "Ineligible"

#MORTSTAT: Final Mortality Status

table(dsn$mortstat, useNA="ifany")

# 0 = Assumed alive

# 1 = Assumed deceased

# <NA> = Ineligible or under age 18

#UCOD_LEADING: Underlying Cause of Death: Recode

table(dsn$ucod_leading, useNA="ifany")

# 1 = Diseases of heart (I00-I09, I11, I13, I20-I51)

# 2 = Malignant neoplasms (C00-C97)

# 3 = Chronic lower respiratory diseases (J40-J47)

# 4 = Accidents (unintentional injuries) (V01-X59, Y85-Y86)

# 5 = Cerebrovascular diseases (I60-I69)

# 6 = Alzheimer's disease (G30)

# 7 = Diabetes mellitus (E10-E14)

# 8 = Influenza and pneumonia (J09-J18)

# 9 = Nephritis, nephrotic syndrome and nephrosis (N00-N07, N17-N19, N25-N27)

# 10 = All other causes (residual)

# <NA> = Ineligible, under age 18, assumed alive, or no cause of death data

#DIABETES: Diabetes Flag from Multiple Cause of Death (MCOD)

table(dsn$diabetes, useNA="ifany")

# 0 = No - Condition not listed as a multiple cause of death

# 1 = Yes - Condition listed as a multiple cause of death

# <NA> = Assumed alive, under age 18, ineligible for mortality follow-up, or MCOD not available

#HYPERTEN: Hypertension Flag from Multiple Cause of Death (MCOD)

table(dsn$hyperten, useNA="ifany")

# 0 = No - Condition not listed as a multiple cause of death

# 1 = Yes - Condition listed as a multiple cause of death

# <NA> = Assumed alive, under age 18, ineligible for mortality follow-up, or MCOD not available

# Re-name the dataset, DSN, to the short survey name then remove other R objects

assign(paste0(srvyout), dsn)

rm(dsn, srvyin, srvyout)

################

#2011#

################

srvyin <- paste("NHANES_2011_2012_MORT_2019_PUBLIC.dat") # full .DAT name here

srvyout <- "nh11mort19" # shorthand dataset name here

# read in the fixed-width format ASCII file

dsn <- read_fwf(file=srvyin,

col_types = "ciiiiiiiddii",

fwf_cols(publicid = c(1,14),

eligstat = c(15,15),

mortstat = c(16,16),

ucod_leading = c(17,19),

diabetes = c(20,20),

hyperten = c(21,21),

dodqtr = c(22,22),

dodyear = c(23,26),

wgt_new = c(27,34),

sa_wgt_new = c(35,42),

permth_int = c(43,45),

permth_exm = c(46,48)

),

na = "."

)

# create the ID (SEQN) for the NHANES surveys

dsn$seqn <- substr(dsn$publicid,1,5)

# NOTE: SEQN is the unique ID for NHANES.

#Drop NHIS variables

dsn <- select(dsn, -publicid)

dsn <- select(dsn, -dodqtr)

dsn <- select(dsn, -dodyear)

dsn <- select(dsn, -wgt_new)

dsn <- select(dsn, -sa_wgt_new)

# Structure and contents of data

str(dsn)

# Variable frequencies

#ELIGSTAT: Eligibility Status for Mortality Follow-up

table(dsn$eligstat)

#1 = "Eligible"

#2 = "Under age 18, not available for public release"

#3 = "Ineligible"

#MORTSTAT: Final Mortality Status

table(dsn$mortstat, useNA="ifany")

# 0 = Assumed alive

# 1 = Assumed deceased

# <NA> = Ineligible or under age 18

#UCOD_LEADING: Underlying Cause of Death: Recode

table(dsn$ucod_leading, useNA="ifany")

# 1 = Diseases of heart (I00-I09, I11, I13, I20-I51)

# 2 = Malignant neoplasms (C00-C97)

# 3 = Chronic lower respiratory diseases (J40-J47)

# 4 = Accidents (unintentional injuries) (V01-X59, Y85-Y86)

# 5 = Cerebrovascular diseases (I60-I69)

# 6 = Alzheimer's disease (G30)

# 7 = Diabetes mellitus (E10-E14)

# 8 = Influenza and pneumonia (J09-J18)

# 9 = Nephritis, nephrotic syndrome and nephrosis (N00-N07, N17-N19, N25-N27)

# 10 = All other causes (residual)

# <NA> = Ineligible, under age 18, assumed alive, or no cause of death data

#DIABETES: Diabetes Flag from Multiple Cause of Death (MCOD)

table(dsn$diabetes, useNA="ifany")

# 0 = No - Condition not listed as a multiple cause of death

# 1 = Yes - Condition listed as a multiple cause of death

# <NA> = Assumed alive, under age 18, ineligible for mortality follow-up, or MCOD not available

#HYPERTEN: Hypertension Flag from Multiple Cause of Death (MCOD)

table(dsn$hyperten, useNA="ifany")

# 0 = No - Condition not listed as a multiple cause of death

# 1 = Yes - Condition listed as a multiple cause of death

# <NA> = Assumed alive, under age 18, ineligible for mortality follow-up, or MCOD not available

# Re-name the dataset, DSN, to the short survey name then remove other R objects

assign(paste0(srvyout), dsn)

rm(dsn, srvyin, srvyout)

################

#2013#

################

srvyin <- paste("NHANES_2013_2014_MORT_2019_PUBLIC.dat") # full .DAT name here

srvyout <- "nh13mort19" # shorthand dataset name here

# read in the fixed-width format ASCII file

dsn <- read_fwf(file=srvyin,

col_types = "ciiiiiiiddii",

fwf_cols(publicid = c(1,14),

eligstat = c(15,15),

mortstat = c(16,16),

ucod_leading = c(17,19),

diabetes = c(20,20),

hyperten = c(21,21),

dodqtr = c(22,22),

dodyear = c(23,26),

wgt_new = c(27,34),

sa_wgt_new = c(35,42),

permth_int = c(43,45),

permth_exm = c(46,48)

),

na = "."

)

# create the ID (SEQN) for the NHANES surveys

dsn$seqn <- substr(dsn$publicid,1,5)

# NOTE: SEQN is the unique ID for NHANES.

#Drop NHIS variables

dsn <- select(dsn, -publicid)

dsn <- select(dsn, -dodqtr)

dsn <- select(dsn, -dodyear)

dsn <- select(dsn, -wgt_new)

dsn <- select(dsn, -sa_wgt_new)

# Structure and contents of data

str(dsn)

# Variable frequencies

#ELIGSTAT: Eligibility Status for Mortality Follow-up

table(dsn$eligstat)

#1 = "Eligible"

#2 = "Under age 18, not available for public release"

#3 = "Ineligible"

#MORTSTAT: Final Mortality Status

table(dsn$mortstat, useNA="ifany")

# 0 = Assumed alive

# 1 = Assumed deceased

# <NA> = Ineligible or under age 18

#UCOD_LEADING: Underlying Cause of Death: Recode

table(dsn$ucod_leading, useNA="ifany")

# 1 = Diseases of heart (I00-I09, I11, I13, I20-I51)

# 2 = Malignant neoplasms (C00-C97)

# 3 = Chronic lower respiratory diseases (J40-J47)

# 4 = Accidents (unintentional injuries) (V01-X59, Y85-Y86)

# 5 = Cerebrovascular diseases (I60-I69)

# 6 = Alzheimer's disease (G30)

# 7 = Diabetes mellitus (E10-E14)

# 8 = Influenza and pneumonia (J09-J18)

# 9 = Nephritis, nephrotic syndrome and nephrosis (N00-N07, N17-N19, N25-N27)

# 10 = All other causes (residual)

# <NA> = Ineligible, under age 18, assumed alive, or no cause of death data

#DIABETES: Diabetes Flag from Multiple Cause of Death (MCOD)

table(dsn$diabetes, useNA="ifany")

# 0 = No - Condition not listed as a multiple cause of death

# 1 = Yes - Condition listed as a multiple cause of death

# <NA> = Assumed alive, under age 18, ineligible for mortality follow-up, or MCOD not available

#HYPERTEN: Hypertension Flag from Multiple Cause of Death (MCOD)

table(dsn$hyperten, useNA="ifany")

# 0 = No - Condition not listed as a multiple cause of death

# 1 = Yes - Condition listed as a multiple cause of death

# <NA> = Assumed alive, under age 18, ineligible for mortality follow-up, or MCOD not available

# Re-name the dataset, DSN, to the short survey name then remove other R objects

assign(paste0(srvyout), dsn)

rm(dsn, srvyin, srvyout)

################

#2015#

################

srvyin <- paste("NHANES_2015_2016_MORT_2019_PUBLIC.dat") # full .DAT name here

srvyout <- "nh15mort19" # shorthand dataset name here

# read in the fixed-width format ASCII file

dsn <- read_fwf(file=srvyin,

col_types = "ciiiiiiiddii",

fwf_cols(publicid = c(1,14),

eligstat = c(15,15),

mortstat = c(16,16),

ucod_leading = c(17,19),

diabetes = c(20,20),

hyperten = c(21,21),

dodqtr = c(22,22),

dodyear = c(23,26),

wgt_new = c(27,34),

sa_wgt_new = c(35,42),

permth_int = c(43,45),

permth_exm = c(46,48)

),

na = "."

)

# create the ID (SEQN) for the NHANES surveys

dsn$seqn <- substr(dsn$publicid,1,5)

# NOTE: SEQN is the unique ID for NHANES.

#Drop NHIS variables

dsn <- select(dsn, -publicid)

dsn <- select(dsn, -dodqtr)

dsn <- select(dsn, -dodyear)

dsn <- select(dsn, -wgt_new)

dsn <- select(dsn, -sa_wgt_new)

# Structure and contents of data

str(dsn)

# Variable frequencies

#ELIGSTAT: Eligibility Status for Mortality Follow-up

table(dsn$eligstat)

#1 = "Eligible"

#2 = "Under age 18, not available for public release"

#3 = "Ineligible"

#MORTSTAT: Final Mortality Status

table(dsn$mortstat, useNA="ifany")

# 0 = Assumed alive

# 1 = Assumed deceased

# <NA> = Ineligible or under age 18

#UCOD_LEADING: Underlying Cause of Death: Recode

table(dsn$ucod_leading, useNA="ifany")

# 1 = Diseases of heart (I00-I09, I11, I13, I20-I51)

# 2 = Malignant neoplasms (C00-C97)

# 3 = Chronic lower respiratory diseases (J40-J47)

# 4 = Accidents (unintentional injuries) (V01-X59, Y85-Y86)

# 5 = Cerebrovascular diseases (I60-I69)

# 6 = Alzheimer's disease (G30)

# 7 = Diabetes mellitus (E10-E14)

# 8 = Influenza and pneumonia (J09-J18)

# 9 = Nephritis, nephrotic syndrome and nephrosis (N00-N07, N17-N19, N25-N27)

# 10 = All other causes (residual)

# <NA> = Ineligible, under age 18, assumed alive, or no cause of death data

#DIABETES: Diabetes Flag from Multiple Cause of Death (MCOD)

table(dsn$diabetes, useNA="ifany")

# 0 = No - Condition not listed as a multiple cause of death

# 1 = Yes - Condition listed as a multiple cause of death

# <NA> = Assumed alive, under age 18, ineligible for mortality follow-up, or MCOD not available

#HYPERTEN: Hypertension Flag from Multiple Cause of Death (MCOD)

table(dsn$hyperten, useNA="ifany")

# 0 = No - Condition not listed as a multiple cause of death

# 1 = Yes - Condition listed as a multiple cause of death

# <NA> = Assumed alive, under age 18, ineligible for mortality follow-up, or MCOD not available

# Re-name the dataset, DSN, to the short survey name then remove other R objects

assign(paste0(srvyout), dsn)

rm(dsn, srvyin, srvyout)

nh3mort19$ucod_leading<-as.character(nh3mort19$ucod_leading)

nh99mort19$ucod_leading<-as.character(nh99mort19$ucod_leading)

nh01mort19$ucod_leading<-as.character(nh01mort19$ucod_leading)

nh03mort19$ucod_leading<-as.character(nh03mort19$ucod_leading)

nh11mort19$ucod_leading<-as.character(nh11mort19$ucod_leading)

nh13mort19$ucod_leading<-as.character(nh13mort19$ucod_leading)

nh15mort19$ucod_leading<-as.character(nh15mort19$ucod_leading)

#2019 MORTALITY FILES

#nh3

nh3mort19$eligstat19 <- nh3mort19$eligstat

nh3mort19$mortstat19 <- nh3mort19$mortstat

nh3mort19$ucod_leading19 <- nh3mort19$ucod_leading

nh3mort19$permth_int19 <- nh3mort19$permth_int

nh3mort19$permth_exm19 <- nh3mort19$permth_exm

#nh99

nh99mort19$eligstat19 <- nh99mort19$eligstat

nh99mort19$mortstat19 <- nh99mort19$mortstat

nh99mort19$ucod_leading19 <- nh99mort19$ucod_leading

nh99mort19$permth_int19 <- nh99mort19$permth_int

nh99mort19$permth_exm19 <- nh99mort19$permth_exm

#nh01

nh01mort19$eligstat19 <- nh01mort19$eligstat

nh01mort19$mortstat19 <- nh01mort19$mortstat

nh01mort19$ucod_leading19 <- nh01mort19$ucod_leading

nh01mort19$permth_int19 <- nh01mort19$permth_int

nh01mort19$permth_exm19 <- nh01mort19$permth_exm

#nh03

nh03mort19$eligstat19 <- nh03mort19$eligstat

nh03mort19$mortstat19 <- nh03mort19$mortstat

nh03mort19$ucod_leading19 <- nh03mort19$ucod_leading

nh03mort19$permth_int19 <- nh03mort19$permth_int

nh03mort19$permth_exm19 <- nh03mort19$permth_exm

#nh11

nh11mort19$eligstat19 <- nh11mort19$eligstat

nh11mort19$mortstat19 <- nh11mort19$mortstat

nh11mort19$ucod_leading19 <- nh11mort19$ucod_leading

nh11mort19$permth_int19 <- nh11mort19$permth_int

nh11mort19$permth_exm19 <- nh11mort19$permth_exm

#nh13

nh13mort19$eligstat19 <- nh13mort19$eligstat

nh13mort19$mortstat19 <- nh13mort19$mortstat

nh13mort19$ucod_leading19 <- nh13mort19$ucod_leading

nh13mort19$permth_int19 <- nh13mort19$permth_int

nh13mort19$permth_exm19 <- nh13mort19$permth_exm

#nh15

nh15mort19$eligstat19 <- nh15mort19$eligstat

nh15mort19$mortstat19 <- nh15mort19$mortstat

nh15mort19$ucod_leading19 <- nh15mort19$ucod_leading

nh15mort19$permth_int19 <- nh15mort19$permth_int

nh15mort19$permth_exm19 <- nh15mort19$permth_exm

nh3mort19$seqn<-as.double(nh3mort19$seqn)

nh99mort19$seqn<-as.double(nh99mort19$seqn)

nh01mort19$seqn<-as.double(nh01mort19$seqn)

nh03mort19$seqn<-as.double(nh03mort19$seqn)

nh11mort19$seqn<-as.double(nh11mort19$seqn)

nh13mort19$seqn<-as.double(nh13mort19$seqn)

nh15mort19$seqn<-as.double(nh15mort19$seqn)

**ANALYSES**

library(foreign)

library(survey)

library(survival)

require(splines)

require(car)

library(readr)

library(writexl)

detach("package:dplyr", unload = TRUE)

setwd("C:/Users/Xaust/Box/NHANES/Updated Mortality Files 2015")

#load demographic and lab data

nh3<-read.dta("MERGENH3_2.dta", convert.factors = F)

nh3<-merge(nh3, nh3mort19, by="seqn")

nh3lab<-read.dta("nh3labs.dta", convert.factors = F)

nh99<-read.dta("MERGENH99_2.dta", convert.factors = F)

nh01<-read.dta("demos01.dta")

names(nh01)<-tolower(names(nh01))

nh01<-merge(nh01, nh01mort19, by="seqn")

nh01test<-read.xport("nh01sexSSCHL_B.XPT") #add mortality to demos

names(nh01test)<-tolower(names(nh01test))

nh01<-merge(nh01, nh01test, by="seqn", all.x=T)

nh03<-read.dta("MERGENH03_2.dta", convert.factors = F)

nh03<-merge(nh03, nh03mort19, by="seqn")

nh3exam<-read.dta("NH3EXAM.dta", convert.factors = F)

nh3<-subset(nh3, eligstat19==1&is.na(sstesto)==F&hfagerr!=888)

nh3<-merge(nh3, nh3lab, by="seqn")

nh3exam$bmi<-ifelse(nh3exam$bmpbmi==8888, NA, nh3exam$bmpbmi)

names(nh3lab)<-tolower(names(nh3lab))

nh3exam<-nh3exam[, c("seqn", "bmi")]

nh3<-merge(nh3, nh3exam, by="seqn", all.x=T)

nh3$crp<-ifelse(nh3$crp>88800, NA, nh3$crp)

nh3$wcp<-ifelse(nh3$wcp==88888, NA, nh3$wcp)

nh3$lympho<-ifelse(nh3$lmppcnt==88888, NA, nh3$lmppcnt)

nh3$mononuc<-ifelse(nh3$moppcnt==88888, NA, nh3$moppcnt)

nh3$granu<-ifelse(nh3$grppcnt==88888, NA, nh3$grppcnt)

nh3$har3<-ifelse(nh3$har3>2,NA,nh3$har3)

nh3$smoke<-nh3$har3

nh3$smoke<-recode(nh3$smoke, recodes="2=0; 1=1; else=NA")

#2019 mortality followup

nh3$year<-ifelse(nh3$sdpphase.x==1, 1990, 1992)

nh3$d.age<-ifelse(nh3$mortstat19==1, nh3$hfagerr+round(nh3$permth_int19/ 12,1), 2019-(nh3$year-nh3$hfagerr))

nh3$timetodeath<-round(nh3$permth_int19/12)

nh3$d.event<-ifelse(nh3$mortstat19==1,1,0)

nh3$d.2yr<-ifelse(nh3$timetodeath<=2,1,0)

nh3$minority<-ifelse(nh3$dmarethn.x>1, 1,0)

nh3$black<-ifelse(nh3$dmarethn.x==2&nh3$dmaethnr.x==3, 1,0)

nh3$white<-ifelse(nh3$dmarethn.x==1&nh3$dmaethnr.x==3, 1,0)

nh3$other<-ifelse(nh3$dmarethn.x==4&nh3$dmaethnr.x==3, 1,0)

nh3$hisp<-ifelse(nh3$dmaethnr.x%in%c(1,2),1,0)

nh3$educ<-recode(nh3$hfa8r, recodes=" 88:99=NA; 0:11=1; 12=2; 13:20=3")

nh3$d_resid<-ifelse(nh3$ucod_leading19=="010", 1, 0)

nh3$psu<-nh3$sdppsu6.x

nh3$strata<-nh3$sdpstra6.x

nh3$pwt<-nh3$wtpfhx6.x

options("survey.lonely.psu"="average")

des<-svydesign(ids = ~sdppsu6.x, strata = ~sdpstra6.x, weights=~wtpfhx6.x, data=nh3, nest=T)

#####Need to add exam files to these other years #other years

nh99crp<-read.xport("nh99crplab.XPT")

names(nh99crp)<-tolower(names(nh99crp))

nh99blood<-read.xport("nh99bloodlab.XPT")

names(nh99blood)<- tolower(names(nh99blood))

nh99bmi<-read.xport("BMX.XPT")

names(nh99bmi)<-tolower(names(nh99bmi))

nh99smoke<-read.xport("SMQ.XPT")

names(nh99smoke)<-tolower(names(nh99smoke))

nh99lab<-merge(nh99crp, nh99blood, by="seqn")

nh99other<-merge(nh99bmi, nh99smoke, by="seqn")

nh99lab<-merge(nh99lab, nh99other, by="seqn")

nh99<-merge(nh99, nh99lab, by="seqn")

nh99<-merge(nh99, nh99mort19, by="seqn")

nh99<-subset(nh99, eligstat19==1&is.na(sstesto)==F)

nh99$crp<-nh99$lbxcrp

nh99$bmi<-nh99$bmxbmi

nh99$smoke<-nh99$smq040

nh99$smoke<-ifelse(nh99$smoke > 3, NA, nh99$smoke)

nh99$smoke<-recode(nh99$smoke, recodes="3=0; 2=1; 1=1; else=NA")

#2019 mortality followup

nh99$year<-1999

nh99$d.age<-ifelse(nh99$mortstat19==1, nh99$ridageyr+round(nh99$permth_int19/12,1), 2019-(nh99$year-nh99$ridageyr))

nh99$timetodeath<-round(nh99$permth_int19/12)

nh99$d.event<-ifelse(nh99$mortstat19==1,1,0)

nh99$d.2yr<-ifelse(nh99$timetodeath<=2,1,0)

nh99$minority<-ifelse(nh99$ridreth1%in%c(1,2,4,5), 1,ifelse(nh99$ridreth1==3, 0, NA))

nh99$black<-ifelse(nh99$ridreth2==4, 1,ifelse(is.na(nh99$ridreth2)==T, NA , 0))

nh99$white<-ifelse(nh99$ridreth2==3, 1,ifelse(is.na(nh99$ridreth2)==T, NA , 0))

nh99$other<-ifelse(nh99$ridreth2==5, 1,ifelse(is.na(nh99$ridreth2)==T, NA , 0))

nh99$hisp<-ifelse(nh99$ridreth2%in%c(1,2), 1,ifelse(is.na(nh99$ridreth2)==T, NA , 0))

nh99$educ<-recode(nh99$dmdeduc, recodes = "1=1; 2=2; 3=3; else=NA")

nh99$d_resid<-ifelse(nh99$ucod_leading19=="010", 1, 0)

nh99$psu<-paste("99",nh99$sdmvpsu, sep="-")

nh99$strata<-paste("99",nh99$sdmvstra, sep="-")

nh99$pwt<-nh99$wtint2yr*.5

options("survey.lonely.psu"="average")

des2<-svydesign(ids = ~sdmvpsu, strata = ~sdmvstra, weights=~wtint2yr, data=nh99, nest=T)

#2001

nh01<-subset(nh01, eligstat19==1&is.na(sstesto)==F)

nh01crp<-read.xport("nh01crplab.XPT")

names(nh01crp)<- tolower(names(nh01crp))

nh01blood<-read.xport("nh01bloodlab.XPT")

names(nh01blood)<-tolower(names(nh01blood))

nh01bmi<-read.xport("BMX_B.XPT")

names(nh01bmi)<-tolower(names(nh01bmi))

nh01smoke<-read.xport("SMQ_B.XPT")

names(nh01smoke)<-tolower(names(nh01smoke))

nh01lab<-merge(nh01crp, nh01blood, by="seqn")

nh01other<-merge(nh01bmi, nh01smoke, by="seqn")

nh01lab<-merge(nh01lab, nh01other, by="seqn")

nh01<-merge(nh01, nh01lab, by="seqn")

nh01$crp<-nh01$lbxcrp

nh01$wcp<-nh01$lbxmopct

nh01$lympho<-nh01$lbxlypct

nh01$mononuc<-nh01$lbxmopct

nh01$bmi<-nh01$bmxbmi

nh01$smoke<-nh01$smq040

nh01$smoke<-ifelse(nh01$smoke > 3, NA, nh01$smoke)

nh01$smoke<-recode(nh01$smoke, recodes="3=0; 2=1; 1=1; else=NA")

#2019 mortality followup

nh01$year<-2001

nh01$d.age<-ifelse(nh01$mortstat19==1, nh01$ridageyr+round(nh01$permth_int19/12,1), 2019-(nh01$year-nh01$ridageyr))

nh01$timetodeath<-round(nh01$permth_int19/12)

nh01$d.event<-ifelse(nh01$mortstat19==1,1,0)

nh01$d.2yr<-ifelse(nh01$timetodeath<=2,1,0)

nh01$minority<-ifelse(nh01$ridreth1%in%c(1,2,4,5), 1,ifelse(nh01$ridreth1==3, 0, NA))

nh01$black<-ifelse(nh01$ridreth2==4, 1,ifelse(is.na(nh01$ridreth2)==T, NA , 0))

nh01$white<-ifelse(nh01$ridreth2==3, 1,ifelse(is.na(nh01$ridreth2)==T, NA , 0))

nh01$other<-ifelse(nh01$ridreth2==5, 1,ifelse(is.na(nh01$ridreth2)==T, NA , 0))

nh01$hisp<-ifelse(nh01$ridreth2%in%c(1,2), 1,ifelse(is.na(nh01$ridreth2)==T, NA , 0))

nh01$educ<-recode(nh01$dmdeduc, recodes = "1=1; 2=2; 3=3; else=NA")

nh01$d_resid<-ifelse(nh01$ucod_leading19=="010", 1, 0)

nh01$psu<-paste("01",nh01$sdmvpsu, sep="-")

nh01$strata<-paste("01",nh01$sdmvstra, sep="-")

nh01$pwt<-nh01$wtint2yr*.5

#2003

nh03crp<-read.xport("nh03crplab.XPT")

names(nh03crp)<-tolower(names(nh03crp))

nh03blood<-read.xport("nh03bloodlab.XPT")

names(nh03blood)<-tolower(names(nh03blood))

nh03bmi<-read.xport("BMX_C.XPT")

names(nh03bmi)<-tolower(names(nh03bmi))

nh03smoke<-read.xport("SMQ_C.XPT")

names(nh03smoke)<-tolower(names(nh03smoke))

nh03lab<-merge(nh03crp, nh03blood, by="seqn")

nh03other<-merge(nh03bmi, nh03smoke, by="seqn")

nh03lab<-merge(nh03lab, nh03other, by="seqn")

nh03<-subset(nh03, eligstat19==1&is.na(sstesto)==F)

nh03<-merge(nh03, nh03lab, by="seqn")

nh03$crp<-nh03$lbxcrp

nh03$wcp<-nh03$lbxmopct

nh03$lympho<-nh03$lbxlypct

nh03$mononuc<-nh03$lbxmopct

nh03$bmi<-nh03$bmxbmi

nh03$smoke<-nh03$smq040

nh03$smoke<-ifelse(nh03$smoke > 3, NA, nh03$smoke)

nh03$smoke<-recode(nh03$smoke, recodes="3=0; 2=1; 1=1; else=NA")

#2019 mortality followup

nh03$year<-2003

nh03$d.age<-ifelse(nh03$mortstat19==1, nh03$ridageyr+round(nh03$permth_int19/12,1), 2019-(nh03$year-nh03$ridageyr))

nh03$timetodeath<-round(nh03$permth_int19/12)

nh03$d.event<-ifelse(nh03$mortstat19==1,1,0)

nh03$d.2yr<-ifelse(nh03$timetodeath<=2,1,0)

nh03$minority<-ifelse(nh03$ridreth1%in%c(1,2,4,5), 1,ifelse(nh03$ridreth1==3, 0, NA))

nh03$black<-ifelse(nh03$ridreth2==4, 1,ifelse(is.na(nh03$ridreth2)==T, NA , 0))

nh03$white<-ifelse(nh03$ridreth2==3, 1,ifelse(is.na(nh03$ridreth2)==T, NA , 0))

nh03$other<-ifelse(nh03$ridreth2==5, 1,ifelse(is.na(nh03$ridreth2)==T, NA , 0))

nh03$hisp<-ifelse(nh03$ridreth2%in%c(1,2), 1,ifelse(is.na(nh03$ridreth2)==T, NA , 0))

nh03$educ<-recode(nh03$dmdeduc, recodes = "1=1; 2=2; 3=3; else=NA")

nh03$d_resid<-ifelse(nh03$ucod_leading19=="010", 1, 0)

nh03$psu<-paste("03",nh03$sdmvpsu, sep="-")

nh03$strata<-paste("03",nh03$sdmvstra, sep="-")

nh03$pwt<-nh03$wtint2yr*.5

#add 2011-2012 data

nh11demo <- read.xport("DEMO_G.XPT")

names(nh11demo)<-tolower(names(nh11demo))

nh11demo <- subset(nh11demo, riagendr==1)

nh11test <-read.xport("TST_G.XPT")

names(nh11test)<-tolower(names(nh11test))

nh11test<-merge(nh11test, nh11mort19, by="seqn")

nh11test<-subset(nh11test,eligstat19==1&is.na(lbxtst)==F)

nh11 <- merge(nh11demo, nh11test, by="seqn")

nh11bmi <-read.xport("BMX_G.XPT")

names(nh11bmi)<-tolower(names(nh11bmi))

nh11<- merge(nh11, nh11bmi, by="seqn")

nh11$bmi<-nh11$bmxbmi

nh11smq <-read.xport("SMQ_G.XPT")

names(nh11smq)<-tolower(names(nh11smq))

nh11smq$smoke<-ifelse(nh11smq$smq040 > 3, NA, nh11smq$smq040)

nh11smq$smoke<-recode(nh11smq$smoke, recodes="3=0; 2=1; 1=1; else=NA")

nh11<-merge(nh11, nh11smq, by="seqn")

nh11$sstesto<-nh11$lbxtst/100

#2019 mortality followup

nh11$year<-2011

nh11$d.age<-ifelse(nh11$mortstat19==1, nh11$ridageyr+round(nh11$permth_int19/12,1), 2019-(nh11$year-nh11$ridageyr))

nh11$timetodeath<-round(nh11$permth_int19/12)

nh11$d.event<-ifelse(nh11$mortstat19==1,1,0)

nh11$d.2yr<-ifelse(nh11$timetodeath<=2,1,0)

nh11$minority<-ifelse(nh11$ridreth1%in%c(1,2,4,5), 1,ifelse(nh11$ridreth1==3, 0, NA))

nh11$black<-ifelse(nh11$ridreth1==4, 1,ifelse(is.na(nh11$ridreth1)==T, NA , 0))

nh11$white<-ifelse(nh11$ridreth1==3, 1,ifelse(is.na(nh11$ridreth1)==T, NA , 0))

nh11$other<-ifelse(nh11$ridreth1==5, 1,ifelse(is.na(nh11$ridreth1)==T, NA , 0))

nh11$hisp<-ifelse(nh11$ridreth1%in%c(1,2), 1,ifelse(is.na(nh11$ridreth1)==T, NA , 0))

nh11$educ<-recode(nh11$dmdeduc2, recodes = "1=1; 2=1; 3=2; 4=3; 5=3; else=NA")

nh11$d_resid<-ifelse(nh11$ucod_leading19=="010", 1, 0)

nh11$psu<-paste("11",nh11$sdmvpsu, sep="-")

nh11$strata<-paste("11",nh11$sdmvstra, sep="-")

nh11$pwt<-nh11$wtint2yr*.5

#add 2013-2014 data

nh13demo <- read.xport("DEMO_H.XPT")

names(nh13demo)<-tolower(names(nh13demo))

nh13demo <- subset(nh13demo, riagendr==1)

nh13test <-read.xport("TST_H.XPT")

names(nh13test)<-tolower(names(nh13test))

nh13test<-merge(nh13test, nh13mort19, by="seqn")

nh13test<-subset(nh13test,eligstat19==1&is.na(lbxtst)==F)

nh13 <- merge(nh13demo, nh13test, by="seqn")

nh13bmi <-read.xport("BMX_H.XPT")

names(nh13bmi)<-tolower(names(nh13bmi))

nh13<- merge(nh13, nh13bmi, by="seqn")

nh13$bmi<-nh13$bmxbmi

nh13smq <-read.xport("SMQ_H.XPT")

names(nh13smq)<-tolower(names(nh13smq))

nh13smq$smoke<-ifelse(nh13smq$smq040 > 3, NA, nh13smq$smq040)

nh13smq$smoke<-recode(nh13smq$smoke, recodes="3=0; 2=1; 1=1; else=NA")

nh13<-merge(nh13, nh13smq, by="seqn")

nh13$sstesto<-nh13$lbxtst/100

#2019 mortality followup

nh13$year<-2013

nh13$d.age<-ifelse(nh13$mortstat19==1, nh13$ridageyr+round(nh13$permth_int19/12,1), 2019-(nh13$year-nh13$ridageyr))

nh13$timetodeath<-round(nh13$permth_int19/12)

nh13$d.event<-ifelse(nh13$mortstat19==1,1,0)

nh13$d.2yr<-ifelse(nh13$timetodeath<=2,1,0)

nh13$minority<-ifelse(nh13$ridreth1%in%c(1,2,4,5), 1,ifelse(nh13$ridreth1==3, 0, NA))

nh13$black<-ifelse(nh13$ridreth1==4, 1,ifelse(is.na(nh13$ridreth1)==T, NA , 0))

nh13$white<-ifelse(nh13$ridreth1==3, 1,ifelse(is.na(nh13$ridreth1)==T, NA , 0))

nh13$other<-ifelse(nh13$ridreth1==5, 1,ifelse(is.na(nh13$ridreth1)==T, NA , 0))

nh13$hisp<-ifelse(nh13$ridreth1%in%c(1,2), 1,ifelse(is.na(nh13$ridreth1)==T, NA , 0))

nh13$educ<-recode(nh13$dmdeduc2, recodes = "1=1; 2=1; 3=2; 4=3; 5=3; else=NA")

nh13$d_resid<-ifelse(nh13$ucod_leading19=="010", 1, 0)

nh13$psu<-paste("13",nh13$sdmvpsu, sep="-")

nh13$strata<-paste("13",nh13$sdmvstra, sep="-")

nh13$pwt<-nh13$wtint2yr*.5

#add 2015-2016 data

nh15demo <- read.xport("DEMO_I.XPT")

names(nh15demo)<-tolower(names(nh15demo))

nh15demo <- subset(nh15demo, riagendr==1)

nh15test <-read.xport("TST_I.XPT")

names(nh15test)<-tolower(names(nh15test))

nh15test<-merge(nh15test, nh15mort19, by="seqn")

nh15test<-subset(nh15test,eligstat19==1&is.na(lbxtst)==F)

nh15 <- merge(nh15demo, nh15test, by="seqn")

nh15bmi <-read.xport("BMX_I.XPT")

names(nh15bmi)<-tolower(names(nh15bmi))

nh15<- merge(nh15, nh15bmi, by="seqn")

nh15$bmi<-nh15$bmxbmi

nh15smq <-read.xport("SMQ_I.XPT")

names(nh15smq)<-tolower(names(nh15smq))

nh15smq$smoke<-ifelse(nh15smq$smq040 > 3, NA, nh15smq$smq040)

nh15smq$smoke<-recode(nh15smq$smoke, recodes="3=0; 2=1; 1=1; else=NA")

nh15<-merge(nh15, nh15smq, by="seqn")

nh15$sstesto<-nh15$lbxtst/100

#2019 mortality followup

nh15$year<-2015

nh15$d.age<-ifelse(nh15$mortstat19==1, nh15$ridageyr+round(nh15$permth_int19/12,1), 2019-(nh15$year-nh15$ridageyr))

nh15$timetodeath<-round(nh15$permth_int19/12)

nh15$d.event<-ifelse(nh15$mortstat19==1,1,0)

nh15$d.2yr<-ifelse(nh15$timetodeath<=2,1,0)

nh15$minority<-ifelse(nh15$ridreth1%in%c(1,2,4,5), 1,ifelse(nh15$ridreth1==3, 0, NA))

nh15$black<-ifelse(nh15$ridreth1==4, 1,ifelse(is.na(nh15$ridreth1)==T, NA , 0))

nh15$white<-ifelse(nh15$ridreth1==3, 1,ifelse(is.na(nh15$ridreth1)==T, NA , 0))

nh15$other<-ifelse(nh15$ridreth1==5, 1,ifelse(is.na(nh15$ridreth1)==T, NA , 0))

nh15$hisp<-ifelse(nh15$ridreth1%in%c(1,2), 1,ifelse(is.na(nh15$ridreth1)==T, NA , 0))

nh15$educ<-recode(nh15$dmdeduc, recodes = "1=1; 2=1; 3=2; 4=3; 5=3; else=NA")

nh15$d_resid<-ifelse(nh15$ucod_leading19=="010", 1, 0)

nh15$psu<-paste("15",nh15$sdmvpsu, sep="-")

nh15$strata<-paste("15",nh15$sdmvstra, sep="-")

nh15$pwt<-nh15$wtint2yr*.5

#SUBSET

#data with CRP

nh3.sub<-nh3[, c("seqn", "year", "d.age", "d.event", "d.2yr","d_resid", "ucod_leading19", "sstesto", "crp", "minority","black","white", "other","hisp", "educ", "psu", "strata", "pwt", "bmi", "smoke")]

nh99.sub<-nh99[, c("seqn","year", "d.age", "d.event", "d.2yr","d_resid", "ucod_leading19", "sstesto", "crp", "minority","black","white", "other","hisp", "educ", "psu", "strata", "pwt", "bmi", "smoke")]

nh01.sub<-nh01[, c("seqn","year", "d.age", "d.event", "d.2yr","d_resid", "ucod_leading19", "sstesto", "crp", "minority","black","white", "other","hisp", "educ", "psu", "strata", "pwt", "bmi", "smoke")]

nh03.sub<-nh03[, c("seqn","year", "d.age", "d.event", "d.2yr","d_resid", "ucod_leading19", "sstesto", "crp", "minority","black","white", "other","hisp", "educ", "psu", "strata", "pwt", "bmi", "smoke")]

#Data without CRP(full)

nh3.sub2<-nh3[, c("seqn", "year", "d.age", "d.event", "d.2yr","d_resid", "ucod_leading19", "sstesto", "minority","black","white", "other","hisp", "educ", "psu", "strata", "pwt", "bmi", "smoke")]

nh99.sub2<-nh99[, c("seqn","year", "d.age", "d.event", "d.2yr","d_resid", "ucod_leading19", "sstesto", "minority","black","white", "other","hisp", "educ", "psu", "strata", "pwt", "bmi", "smoke")]

nh01.sub2<-nh01[, c("seqn","year", "d.age", "d.event", "d.2yr","d_resid", "ucod_leading19", "sstesto", "minority","black","white", "other","hisp", "educ", "psu", "strata", "pwt", "bmi", "smoke")]

nh03.sub2<-nh03[, c("seqn","year", "d.age", "d.event", "d.2yr","d_resid", "ucod_leading19", "sstesto", "minority","black","white", "other","hisp", "educ", "psu", "strata", "pwt", "bmi", "smoke")]

nh11.sub<-nh11[, c("seqn","year", "d.age", "d.event", "d.2yr","d_resid", "ucod_leading19", "sstesto", "minority","black","white", "other","hisp", "educ", "psu", "strata", "pwt", "bmi", "smoke")]

nh13.sub<-nh13[, c("seqn","year", "d.age", "d.event", "d.2yr","d_resid", "ucod_leading19", "sstesto", "minority","black","white", "other","hisp", "educ", "psu", "strata", "pwt", "bmi", "smoke")]

nh15.sub<-nh15[, c("seqn","year", "d.age", "d.event", "d.2yr","d_resid", "ucod_leading19", "sstesto", "minority","black","white", "other","hisp", "educ", "psu", "strata", "pwt", "bmi", "smoke")]

nh_all_merge<-rbind(nh3.sub, nh99.sub, nh01.sub, nh03.sub)

nh_all_merge2<-rbind(nh3.sub2, nh99.sub2, nh01.sub2, nh03.sub2, nh11.sub, nh13.sub, nh15.sub)

nh_all_merge$ucod_leading19<-ifelse(is.na(nh_all_merge$ucod_leading19),0,nh_all_merge$ucod_leading19)

nh_all_merge2$ucod_leading19<-ifelse(is.na(nh_all_merge2$ucod_leading19),0,nh_all_merge2$ucod_leading19)

#data with crp

nh_all_merge$die_01<-recode(nh_all_merge$ucod_leading19, recodes="0=0;1=1;else=NA")

nh_all_merge$die_02<-recode(nh_all_merge$ucod_leading19, recodes="0=0;2=1;else=NA")

nh_all_merge$die_03<-recode(nh_all_merge$ucod_leading19, recodes="0=0;3=1;else=NA")

nh_all_merge$die_04<-recode(nh_all_merge$ucod_leading19, recodes="0=0;4=1;else=NA")

nh_all_merge$die_05<-recode(nh_all_merge$ucod_leading19, recodes="0=0;5=1;else=NA")

nh_all_merge$die_06<-recode(nh_all_merge$ucod_leading19, recodes="0=0;6=1;else=NA")

nh_all_merge$die_07<-recode(nh_all_merge$ucod_leading19, recodes="0=0;7=1;else=NA")

nh_all_merge$die_08<-recode(nh_all_merge$ucod_leading19, recodes="0=0;8=1;else=NA")

nh_all_merge$die_09<-recode(nh_all_merge$ucod_leading19, recodes="0=0;9=1;else=NA")

nh_all_merge$die_10<-recode(nh_all_merge$ucod_leading19, recodes="0=0;10=1;else=NA")

nh_all_merge$liveorcens<-ifelse(nh_all_merge$ucod_leading19=="0", 1,0)

nh_all_merge$die_all<-recode(nh_all_merge$ucod_leading19, recodes="0=0;1=1;;2=1;3=1;5=1;6=1;7=1;8=1;9=1;else=NA")

#data without crp(full)

nh_all_merge2$die_01<-recode(nh_all_merge2$ucod_leading19, recodes="0=0;1=1;else=NA")

nh_all_merge2$die_02<-recode(nh_all_merge2$ucod_leading19, recodes="0=0;2=1;else=NA")

nh_all_merge2$die_03<-recode(nh_all_merge2$ucod_leading19, recodes="0=0;3=1;else=NA")

nh_all_merge2$die_04<-recode(nh_all_merge2$ucod_leading19, recodes="0=0;4=1;else=NA")

nh_all_merge2$die_05<-recode(nh_all_merge2$ucod_leading19, recodes="0=0;5=1;else=NA")

nh_all_merge2$die_06<-recode(nh_all_merge2$ucod_leading19, recodes="0=0;6=1;else=NA")

nh_all_merge2$die_07<-recode(nh_all_merge2$ucod_leading19, recodes="0=0;7=1;else=NA")

nh_all_merge2$die_08<-recode(nh_all_merge2$ucod_leading19, recodes="0=0;8=1;else=NA")

nh_all_merge2$die_09<-recode(nh_all_merge2$ucod_leading19, recodes="0=0;9=1;else=NA")

nh_all_merge2$die_10<-recode(nh_all_merge2$ucod_leading19, recodes="0=0;10=1;else=NA")

nh_all_merge2$liveorcens<-ifelse(nh_all_merge2$ucod_leading19=="0", 1,0)

nh_all_merge2$die_all<-recode(nh_all_merge2$ucod_leading19, recodes="0=0;1=1;;2=1;3=1;5=1;6=1;7=1;8=1;9=1;else=NA")

#data with CRP: 2019 mortality updated

head(nh_all_merge)

table(nh_all_merge$ucod_leading19)

summary(nh_all_merge$d.age)

nh_all_merge$age_death<-nh_all_merge$d.age

nh_all_merge.pp<-survSplit(nh_all_merge, cut=seq(20, 100, 10), start="start", end="age_death", event="d_resid")

nh_all_merge.pp<-nh_all_merge.pp[order(nh_all_merge.pp$seqn, nh_all_merge.pp$year, nh_all_merge.pp$start),]

head(nh_all_merge.pp, n=20)

des.1<-svydesign(ids=~psu, strata = ~strata, weights=~pwt, data=nh_all_merge, nest=T)

des.m<-svydesign(ids=~psu, strata = ~strata, weights=~pwt, data=nh_all_merge.pp, nest=T)

head(nh_all_merge.pp)

svyby(~sstesto+crp+white+black+other+hisp+educ+d.age+bmi, ~die_10, des.1, svymean, na.rm=T, keep.var = F)

svyby(~sstesto+crp+white+black+other+hisp+educ+d.age+bmi, ~die_09, des.1, svymean, na.rm=T, keep.var = F)

svyby(~sstesto+crp+white+black+other+hisp+educ+d.age+bmi, ~die_08, des.1, svymean, na.rm=T, keep.var = F)

svyby(~sstesto+crp+white+black+other+hisp+educ+d.age+bmi, ~die_07, des.1, svymean, na.rm=T, keep.var = F)

svyby(~sstesto+crp+white+black+other+hisp+educ+d.age+bmi, ~die_06, des.1, svymean, na.rm=T, keep.var = F)

svyby(~sstesto+crp+white+black+other+hisp+educ+d.age+bmi, ~die_05, des.1, svymean, na.rm=T, keep.var = F)

svyby(~sstesto+crp+white+black+other+hisp+educ+d.age+bmi, ~die_04, des.1, svymean, na.rm=T, keep.var = F)

svyby(~sstesto+crp+white+black+other+hisp+educ+d.age+bmi, ~die_03, des.1, svymean, na.rm=T, keep.var = F)

svyby(~sstesto+crp+white+black+other+hisp+educ+d.age+bmi, ~die_02, des.1, svymean, na.rm=T, keep.var = F)

svyby(~sstesto+crp+white+black+other+hisp+educ+d.age+bmi, ~die_01, des.1, svymean, na.rm=T, keep.var = F)

svyby(~sstesto+crp+white+black+other+hisp+educ+d.age+bmi, ~liveorcens, des.1, svymean, na.rm=T, keep.var = F)

sqrt(coef(svyby(~sstesto, ~liveorcens, des.1, svyvar, na.rm=T, keep.var = F)))

sqrt(coef(svyby(~crp, ~liveorcens, des.1, svyvar, na.rm=T, keep.var = F)))

sqrt(coef(svyby(~d.age, ~liveorcens, des.1, svyvar, na.rm=T, keep.var = F)))

sqrt(coef(svyby(~bmi, ~liveorcens, des.1, svyvar, na.rm=T, keep.var = F)))

sqrt(coef(svyby(~educ, ~liveorcens, des.1, svyvar, na.rm=T, keep.var = F)))

sqrt(coef(svyby(~sstesto, ~die_01, des.1, svyvar, na.rm=T, keep.var = F)))

sqrt(coef(svyby(~crp, ~die_01, des.1, svyvar, na.rm=T, keep.var = F)))

sqrt(coef(svyby(~d.age, ~die_01, des.1, svyvar, na.rm=T, keep.var = F)))

sqrt(coef(svyby(~bmi, ~die_01, des.1, svyvar, na.rm=T, keep.var = F)))

sqrt(coef(svyby(~educ, ~die_01, des.1, svyvar, na.rm=T, keep.var = F)))

sqrt(coef(svyby(~sstesto, ~die_02, des.1, svyvar, na.rm=T, keep.var = F)))

sqrt(coef(svyby(~crp, ~die_02, des.1, svyvar, na.rm=T, keep.var = F)))

sqrt(coef(svyby(~d.age, ~die_02, des.1, svyvar, na.rm=T, keep.var = F)))

sqrt(coef(svyby(~bmi, ~die_02, des.1, svyvar, na.rm=T, keep.var = F)))

sqrt(coef(svyby(~educ, ~die_02, des.1, svyvar, na.rm=T, keep.var = F)))

sqrt(coef(svyby(~sstesto, ~die_03, des.1, svyvar, na.rm=T, keep.var = F)))

sqrt(coef(svyby(~crp, ~die_03, des.1, svyvar, na.rm=T, keep.var = F)))

sqrt(coef(svyby(~d.age, ~die_03, des.1, svyvar, na.rm=T, keep.var = F)))

sqrt(coef(svyby(~bmi, ~die_03, des.1, svyvar, na.rm=T, keep.var = F)))

sqrt(coef(svyby(~educ, ~die_03, des.1, svyvar, na.rm=T, keep.var = F)))

sqrt(coef(svyby(~sstesto, ~die_04, des.1, svyvar, na.rm=T, keep.var = F)))

sqrt(coef(svyby(~crp, ~die_04, des.1, svyvar, na.rm=T, keep.var = F)))

sqrt(coef(svyby(~d.age, ~die_04, des.1, svyvar, na.rm=T, keep.var = F)))

sqrt(coef(svyby(~bmi, ~die_04, des.1, svyvar, na.rm=T, keep.var = F)))

sqrt(coef(svyby(~educ, ~die_04, des.1, svyvar, na.rm=T, keep.var = F)))

sqrt(coef(svyby(~sstesto, ~die_05, des.1, svyvar, na.rm=T, keep.var = F)))

sqrt(coef(svyby(~crp, ~die_05, des.1, svyvar, na.rm=T, keep.var = F)))

sqrt(coef(svyby(~d.age, ~die_05, des.1, svyvar, na.rm=T, keep.var = F)))

sqrt(coef(svyby(~bmi, ~die_05, des.1, svyvar, na.rm=T, keep.var = F)))

sqrt(coef(svyby(~educ, ~die_05, des.1, svyvar, na.rm=T, keep.var = F)))

sqrt(coef(svyby(~sstesto, ~die_06, des.1, svyvar, na.rm=T, keep.var = F)))

sqrt(coef(svyby(~crp, ~die_06, des.1, svyvar, na.rm=T, keep.var = F)))

sqrt(coef(svyby(~d.age, ~die_06, des.1, svyvar, na.rm=T, keep.var = F)))

sqrt(coef(svyby(~bmi, ~die_06, des.1, svyvar, na.rm=T, keep.var = F)))

sqrt(coef(svyby(~educ, ~die_06, des.1, svyvar, na.rm=T, keep.var = F)))

sqrt(coef(svyby(~sstesto, ~die_07, des.1, svyvar, na.rm=T, keep.var = F)))

sqrt(coef(svyby(~crp, ~die_07, des.1, svyvar, na.rm=T, keep.var = F)))

sqrt(coef(svyby(~d.age, ~die_07, des.1, svyvar, na.rm=T, keep.var = F)))

sqrt(coef(svyby(~bmi, ~die_07, des.1, svyvar, na.rm=T, keep.var = F)))

sqrt(coef(svyby(~educ, ~die_07, des.1, svyvar, na.rm=T, keep.var = F)))

sqrt(coef(svyby(~sstesto, ~die_08, des.1, svyvar, na.rm=T, keep.var = F)))

sqrt(coef(svyby(~crp, ~die_08, des.1, svyvar, na.rm=T, keep.var = F)))

sqrt(coef(svyby(~d.age, ~die_08, des.1, svyvar, na.rm=T, keep.var = F)))

sqrt(coef(svyby(~bmi, ~die_08, des.1, svyvar, na.rm=T, keep.var = F)))

sqrt(coef(svyby(~educ, ~die_08, des.1, svyvar, na.rm=T, keep.var = F)))

sqrt(coef(svyby(~sstesto, ~die_09, des.1, svyvar, na.rm=T, keep.var = F)))

sqrt(coef(svyby(~crp, ~die_09, des.1, svyvar, na.rm=T, keep.var = F)))

sqrt(coef(svyby(~d.age, ~die_09, des.1, svyvar, na.rm=T, keep.var = F)))

sqrt(coef(svyby(~bmi, ~die_09, des.1, svyvar, na.rm=T, keep.var = F)))

sqrt(coef(svyby(~educ, ~die_09, des.1, svyvar, na.rm=T, keep.var = F)))

sqrt(coef(svyby(~sstesto, ~die_10, des.1, svyvar, na.rm=T, keep.var = F)))

sqrt(coef(svyby(~crp, ~die_10, des.1, svyvar, na.rm=T, keep.var = F)))

sqrt(coef(svyby(~d.age, ~die_10, des.1, svyvar, na.rm=T, keep.var = F)))

sqrt(coef(svyby(~bmi, ~die_10, des.1, svyvar, na.rm=T, keep.var = F)))

sqrt(coef(svyby(~educ, ~die_10, des.1, svyvar, na.rm=T, keep.var = F)))

svyttest(sstesto~die_01, des.1)

svyttest(sstesto~die_02, des.1)

svyttest(sstesto~die_03, des.1)

svyttest(sstesto~die_04, des.1)

svyttest(sstesto~die_05, des.1)

svyttest(sstesto~die_06, des.1)

svyttest(sstesto~die_07, des.1)

svyttest(sstesto~die_08, des.1)

svyttest(sstesto~die_09, des.1)

svyttest(sstesto~die_10, des.1)

svyttest(crp~die_01, des.1)

svyttest(crp~die_02, des.1)

svyttest(crp~die_03, des.1)

svyttest(crp~die_04, des.1)

svyttest(crp~die_05, des.1)

svyttest(crp~die_06, des.1)

svyttest(crp~die_07, des.1)

svyttest(crp~die_08, des.1)

svyttest(crp~die_09, des.1)

svyttest(crp~die_10, des.1)

svyttest(d.age~die_01, des.1)

svyttest(d.age~die_02, des.1)

svyttest(d.age~die_03, des.1)

svyttest(d.age~die_04, des.1)

svyttest(d.age~die_05, des.1)

svyttest(d.age~die_06, des.1)

svyttest(d.age~die_07, des.1)

svyttest(d.age~die_08, des.1)

svyttest(d.age~die_09, des.1)

svyttest(d.age~die_10, des.1)

svyttest(white~die_01, des.1)

svyttest(white~die_02, des.1)

svyttest(white~die_03, des.1)

svyttest(white~die_04, des.1)

svyttest(white~die_05, des.1)

svyttest(white~die_06, des.1)

svyttest(white~die_07, des.1)

svyttest(white~die_08, des.1)

svyttest(white~die_09, des.1)

svyttest(white~die_10, des.1)

svyttest(black~die_01, des.1)

svyttest(black~die_02, des.1)

svyttest(black~die_03, des.1)

svyttest(black~die_04, des.1)

svyttest(black~die_05, des.1)

svyttest(black~die_06, des.1)

svyttest(black~die_07, des.1)

svyttest(black~die_08, des.1)

svyttest(black~die_09, des.1)

svyttest(black~die_10, des.1)

svyttest(other~die_01, des.1)

svyttest(other~die_02, des.1)

svyttest(other~die_03, des.1)

svyttest(other~die_04, des.1)

svyttest(other~die_05, des.1)

svyttest(other~die_06, des.1)

svyttest(other~die_07, des.1)

svyttest(other~die_08, des.1)

svyttest(other~die_09, des.1)

svyttest(other~die_10, des.1)

svyttest(hisp~die_01, des.1)

svyttest(hisp~die_02, des.1)

svyttest(hisp~die_03, des.1)

svyttest(hisp~die_04, des.1)

svyttest(hisp~die_05, des.1)

svyttest(hisp~die_06, des.1)

svyttest(hisp~die_07, des.1)

svyttest(hisp~die_08, des.1)

svyttest(hisp~die_09, des.1)

svyttest(hisp~die_10, des.1)

svyttest(educ~die_01, des.1)

svyttest(educ~die_02, des.1)

svyttest(educ~die_03, des.1)

svyttest(educ~die_04, des.1)

svyttest(educ~die_05, des.1)

svyttest(educ~die_06, des.1)

svyttest(educ~die_07, des.1)

svyttest(educ~die_08, des.1)

svyttest(educ~die_09, des.1)

svyttest(educ~die_10, des.1)

svyttest(bmi~die_10, des.1)

svyttest(bmi~die_09, des.1)

svyttest(bmi~die_08, des.1)

svyttest(bmi~die_07, des.1)

svyttest(bmi~die_06, des.1)

svyttest(bmi~die_05, des.1)

svyttest(bmi~die_04, des.1)

svyttest(bmi~die_03, des.1)

svyttest(bmi~die_02, des.1)

svyttest(bmi~die_01, des.1)

#binomial model

#heart disease

svglm1<-svyglm(I(die_01)~bs(start)*(sstesto)+crp+black+other+hisp+educ+bmi, des.m, family=binomial(link="cloglog"))

summary(svglm1)

round(cbind(exp(confint(svglm1))[,1], exp(coef(svglm1)), exp(confint(svglm1))[,2]), 2)

#cancer

svglm2<-svyglm(I(die_02)~bs(start)*(sstesto)+crp+black+other+hisp+educ+bmi, des.m, family=binomial(link="cloglog"))

summary(svglm2)

round(cbind(exp(confint(svglm2))[,1], exp(coef(svglm2)), exp(confint(svglm2))[,2]), 2)

#chronic lower resp

svglm3<-svyglm(I(die_03)~bs(start)*(sstesto)+crp+black+other+hisp+educ+bmi, des.m, family=binomial(link="cloglog"))

summary(svglm3)

round(cbind(exp(confint(svglm3))[,1], exp(coef(svglm3)), exp(confint(svglm3))[,2]), 2)

#accidents

svglm4<-svyglm(I(die_04)~bs(start)*(sstesto)+crp+black+other+hisp+educ+bmi, des.m, family=binomial(link="cloglog"))

summary(svglm4)

round(cbind(exp(confint(svglm4))[,1], exp(coef(svglm4)), exp(confint(svglm4))[,2]), 2)

#cerebrovascular

svglm5<-svyglm(I(die_05)~bs(start)*(sstesto)+crp+black+other+hisp+educ+bmi, des.m, family=binomial(link="cloglog"))

summary(svglm5)

round(cbind(exp(confint(svglm5))[,1], exp(coef(svglm5)), exp(confint(svglm5))[,2]), 2)

#alzheimer's

svglm6<-svyglm(I(die_06)~bs(start)*(sstesto)+crp+black+other+hisp+educ+bmi, des.m, family=binomial(link="cloglog"))

summary(svglm6)

round(cbind(exp(confint(svglm6))[,1], exp(coef(svglm6)), exp(confint(svglm6))[,2]), 2)

#diabetes

svglm7<-svyglm(I(die_07)~bs(start)*(sstesto)+crp+black+other+hisp+educ+bmi, des.m, family=binomial(link="cloglog"))

summary(svglm7)

round(cbind(exp(confint(svglm7))[,1], exp(coef(svglm7)), exp(confint(svglm7))[,2]), 2)

#flu and pneumonia

svglm8<-svyglm(I(die_08)~bs(start)*(sstesto)+crp+black+other+hisp+educ+bmi, des.m, family=binomial(link="cloglog"))

summary(svglm8)

round(cbind(exp(confint(svglm8))[,1], exp(coef(svglm8)), exp(confint(svglm8))[,2]), 2)

#kidney disease

svglm9<-svyglm(I(die_09)~bs(start)*(sstesto)+crp+black+other+hisp+educ+bmi, des.m, family=binomial(link="cloglog"))

summary(svglm9)

round(cbind(exp(confint(svglm9))[,1], exp(coef(svglm9)), exp(confint(svglm9))[,2]), 2)

#all cause

svglmall<-svyglm(I(die_all)~bs(start)*(sstesto)+crp+black+other+hisp+educ+bmi, des.m, family=binomial(link="cloglog"))

summary(svglmall)

round(cbind(exp(confint(svglmall))[,1], exp(coef(svglmall)), exp(confint(svglmall))[,2]), 2)

dat<-expand.grid(start=seq(0,100,10),crp=mean(nh_all_merge$crp, na.rm= T), sstesto=quantile(nh_all_merge$sstesto, na.rm=T)[2:4], black=mean(nh_all_merge$black), other=mean(nh_all_merge$other),hisp=mean(nh_all_merge$hisp), educ=mean(nh_all_merge$educ, na.rm= T),bmi=mean(nh_all_merge$bmi, na.rm= T))

dat$fitted<-as.numeric(predict(svglm1, type = "response", newdata=dat)) #heart

dat$fitted2<-as.numeric(predict(svglm2, type = "response", newdata=dat)) #cancer

dat$fitted3<-as.numeric(predict(svglm3, type = "response", newdata=dat)) #lower respiratory

dat$fitted4<-as.numeric(predict(svglm4, type = "response", newdata=dat)) #accidents

dat$fitted5<-as.numeric(predict(svglm5, type = "response", newdata=dat)) #cerebrovascular

dat$fitted6<-as.numeric(predict(svglm6, type = "response", newdata=dat)) #alzheimer's

dat$fitted7<-as.numeric(predict(svglm7, type = "response", newdata=dat)) #diabetes

dat$fitted8<-as.numeric(predict(svglm8, type = "response", newdata=dat)) #influenza and pneumonia

dat$fitted9<-as.numeric(predict(svglm9, type = "response", newdata=dat)) #nephritis

dat$fittedall<-as.numeric(predict(svglmall, type = "response", newdata=dat)) #allcause

dat$age<-as.numeric(as.character(dat$start))

head(dat)

png("C:/Users/Xaust/Box/NHANES/Updated Mortality Files 2015/Figure1b.png", width=2400, height=1600, res = 150)

par(mar = c(5.1,4.1,4.1,2.1)+0.5)

#all cause

plot(dat$age[1:11], dat$fittedall[1:11], yaxt = "n", type="b", ylab="Hazard",lwd=2.5, xlab="Age", col="red", cex.lab = 2, cex.axis = 1.75, cex.main = 2, main =c("All Cause Mortality"), ylim=c(0, 0.8))

points(dat$age[23:33], dat$fittedall[23:33], type="b", col="blue", lwd=2.5)

legend("topleft", legend=c("Low Testosterone", "High Testosterone"), col=c("red", "blue"),cex = 1.75, lwd=1.75,lty=1)

axis(2, at = c(0, 0.2, 0.4, 0.6, 0.8), cex.axis = 1.75)

dev.off()

png("C:/Users/Xaust/Box/NHANES/Updated Mortality Files 2015/Figure2b.png", width=1600, height=2400, res = 150)

par(mfrow=c(3,1), mar = c(5,8,5,5) + 0.1)

#heart

plot(dat$age[1:11], dat$fitted[1:11], yaxt = "n", type="b", ylab="Hazard",lwd=2.5, xlab="Age", col="red", cex.lab = 2,cex.axis = 1.75, cex.main = 2, main =c("Diseases of the Heart"), ylim=c(0, .32))

points(dat$age[23:33], dat$fitted[23:33], type="b",lwd=2.5, col="blue")

legend("topleft", legend=c("Low Testosterone", "High Testosterone"), col=c("red", "blue"), cex = 1.75,lwd=2,lty=1)

axis(2, at = c(0, 0.08, 0.16, 0.24, 0.32), cex.axis = 1.75)

#Cerebrovascular disease

plot(dat$age[1:11], dat$fitted5[1:11], yaxt = "n", type="b", ylab="Hazard",lwd=2.5, xlab="Age", col="red", cex.lab = 2, cex.axis = 1.75, cex.main = 2, main =c("Cerebrovascular Disease"), ylim=c(0, .048))

points(dat$age[23:33], dat$fitted5[23:33], type="b", col="blue", lwd=2.5)

legend("topleft", legend=c("Low Testosterone", "High Testosterone"), col=c("red", "blue"),cex = 1.75, lwd=1.75,lty=1)

axis(2, at = c(0, 0.012, 0.024, 0.036, 0.048), cex.axis = 1.75)

#cancer

plot(dat$age[1:11], dat$fitted2[1:11], yaxt = "n", type="b", ylab="Hazard",lwd=2.5, xlab="Age", col="red", cex.lab = 2, cex.axis = 1.75, cex.main = 2, main =c("Malignant Neoplasms"), ylim=c(0, .32))

points(dat$age[23:33], dat$fitted2[23:33], type="b", col="blue", lwd=2.5)

legend("topleft", legend=c("Low Testosterone", "High Testosterone"), col=c("red", "blue"),cex = 1.75, lwd=1.75,lty=1)

axis(2, at = c(0, 0.08, 0.16, 0.24, 0.32), cex.axis = 1.75)

dev.off()

png("C:/Users/Xaust/Box/NHANES/Updated Mortality Files 2015/Figure3b.png", width=1600, height=2400, res = 150)

par(mfrow=c(3,1), mar = c(5,8,5,5) + 0.1)

#influenza

plot(dat$age[1:11], dat$fitted8[1:11], yaxt = "n", type="b", ylab="Hazard",lwd=2.5, xlab="Age", col="red", cex.lab = 2, cex.axis = 1.75, cex.main = 2, main =c("Influenza and Pneumonia"), ylim=c(0, .016))

points(dat$age[23:33], dat$fitted8[23:33], type="b", col="blue", lwd=2.5)

legend("topleft", legend=c("Low Testosterone", "High Testosterone"), col=c("red", "blue"),cex = 1.75, lwd=1.75,lty=1)

axis(2, at = c(0, 0.004, 0.008, 0.012, 0.016), cex.axis = 1.75)

#chronic respiratory

plot(dat$age[1:11], dat$fitted3[1:11], yaxt = "n", type="b", ylab="Hazard",lwd=2.5, xlab="Age", col="red", cex.lab = 2, cex.axis = 1.75, cex.main = 2, main =c("Chronic Lower Respiratory Diseases"), ylim=c(0, .048))

points(dat$age[23:33], dat$fitted3[23:33], type="b", col="blue", lwd=2.5)

legend("topleft", legend=c("Low Testosterone", "High Testosterone"), col=c("red", "blue"),cex = 1.75, lwd=1.75,lty=1)

axis(2, at = c(0, 0.012, 0.024, 0.036, 0.048), cex.axis = 1.75)

#alzheimers

plot(dat$age[1:11], dat$fitted6[1:11], yaxt = "n", type="b", ylab="Hazard",lwd=2.5, xlab="Age", col="red", cex.lab = 2, cex.axis = 1.75, cex.main = 2, main =c("Alzheimer's Disease"), ylim=c(0, .028))

points(dat$age[23:33], dat$fitted6[23:33], type="b", col="blue", lwd=2.5)

legend("topleft", legend=c("Low Testosterone", "High Testosterone"), col=c("red", "blue"),cex = 1.75, lwd=1.75,lty=1)

axis(2, at = c(0, 0.007, 0.014, 0.021, 0.028), cex.axis = 1.75)

dev.off()

png("C:/Users/Xaust/Box/NHANES/Updated Mortality Files 2015/Figure4b.png", width=1600, height=2400, res = 150)

par(mfrow=c(3,1), mar = c(5,8,5,5) + 0.1)

#accidents

plot(dat$age[1:11], dat$fitted4[1:11], yaxt = "n", type="b", ylab="Hazard",lwd=2.5, xlab="Age", col="red", cex.lab = 2, cex.axis = 1.75, cex.main = 2, main =c("Accidents and Injuries"), ylim=c(0, .024))

points(dat$age[23:33], dat$fitted4[23:33], type="b", col="blue", lwd=2.5)

legend("topleft", legend=c("Low Testosterone", "High Testosterone"), col=c("red", "blue"),cex = 1.75, lwd=1.75,lty=1)

axis(2, at = c(0, 0.006, 0.012, 0.018, 0.024), cex.axis = 1.75)

#diabetes

plot(dat$age[1:11], dat$fitted7[1:11], yaxt = "n", type="b", ylab="Hazard",lwd=2.5, xlab="Age", col="red", cex.lab = 2, cex.axis = 1.75, cex.main = 2, main =c("Diabetes Mellitus"), ylim=c(0, .012))

points(dat$age[23:33], dat$fitted7[23:33], type="b", col="blue", lwd=2.5)

legend("topleft", legend=c("Low Testosterone", "High Testosterone"), col=c("red", "blue"),cex = 1.75, lwd=1.75,lty=1)

axis(2, at = c(0, 0.003, 0.006, 0.009, 0.012), cex.axis = 1.75)

#Nephritis

plot(dat$age[1:11], dat$fitted9[1:11], yaxt = "n", type="b", ylab="Hazard",lwd=2.5, xlab="Age", col="red", cex.lab = 2, cex.axis = 1.75, cex.main = 2, main =c("Kidney Diseases"), ylim=c(0, .016))

points(dat$age[23:33], dat$fitted9[23:33], type="b", col="blue", lwd=2.5)

legend("topleft", legend=c("Low Testosterone", "High Testosterone"), col=c("red", "blue"),cex = 1.75, lwd=1.75,lty=1)

axis(2, at = c(0, 0.004, 0.008, 0.012, 0.016), cex.axis = 1.75)

dev.off()

#Data without CRP (full)

head(nh_all_merge2)

table(nh_all_merge2$ucod_leading19)

summary(nh_all_merge2$d.age)

nh_all_merge2$age_death<-nh_all_merge2$d.age

nh_all_merge2.pp<-survSplit(nh_all_merge2, cut=seq(20, 100, 10), start="start", end="age_death", event="d_resid")

nh_all_merge2.pp<-nh_all_merge2.pp[order(nh_all_merge2.pp$seqn, nh_all_merge2.pp$year, nh_all_merge2.pp$start),]

head(nh_all_merge2.pp, n=20)

des.2<-svydesign(ids=~psu, strata = ~strata, weights=~pwt, data=nh_all_merge2, nest=T)

des.m2<-svydesign(ids=~psu, strata = ~strata, weights=~pwt, data=nh_all_merge2.pp, nest=T)

head(nh_all_merge2.pp)

svyby(~sstesto+white+black+other+hisp+educ+d.age+bmi, ~die_10, des.2, svymean, na.rm=T, keep.var = F)

svyby(~sstesto+white+black+other+hisp+educ+d.age+bmi, ~die_09, des.2, svymean, na.rm=T, keep.var = F)

svyby(~sstesto+white+black+other+hisp+educ+d.age+bmi, ~die_08, des.2, svymean, na.rm=T, keep.var = F)

svyby(~sstesto+white+black+other+hisp+educ+d.age+bmi, ~die_07, des.2, svymean, na.rm=T, keep.var = F)

svyby(~sstesto+white+black+other+hisp+educ+d.age+bmi, ~die_06, des.2, svymean, na.rm=T, keep.var = F)

svyby(~sstesto+white+black+other+hisp+educ+d.age+bmi, ~die_05, des.2, svymean, na.rm=T, keep.var = F)

svyby(~sstesto+white+black+other+hisp+educ+d.age+bmi, ~die_04, des.2, svymean, na.rm=T, keep.var = F)

svyby(~sstesto+white+black+other+hisp+educ+d.age+bmi, ~die_03, des.2, svymean, na.rm=T, keep.var = F)

svyby(~sstesto+white+black+other+hisp+educ+d.age+bmi, ~die_02, des.2, svymean, na.rm=T, keep.var = F)

svyby(~sstesto+white+black+other+hisp+educ+d.age+bmi, ~die_01, des.2, svymean, na.rm=T, keep.var = F)

svyby(~sstesto+white+black+other+hisp+educ+d.age+bmi, ~liveorcens, des.2, svymean, na.rm=T, keep.var = F)

sqrt(coef(svyby(~sstesto, ~liveorcens, des.2, svyvar, na.rm=T, keep.var = F)))

sqrt(coef(svyby(~d.age, ~liveorcens, des.2, svyvar, na.rm=T, keep.var = F)))

sqrt(coef(svyby(~bmi, ~liveorcens, des.2, svyvar, na.rm=T, keep.var = F)))

sqrt(coef(svyby(~educ, ~liveorcens, des.2, svyvar, na.rm=T, keep.var = F)))

sqrt(coef(svyby(~sstesto, ~die_01, des.2, svyvar, na.rm=T, keep.var = F)))

sqrt(coef(svyby(~d.age, ~die_01, des.2, svyvar, na.rm=T, keep.var = F)))

sqrt(coef(svyby(~bmi, ~die_01, des.2, svyvar, na.rm=T, keep.var = F)))

sqrt(coef(svyby(~educ, ~die_01, des.2, svyvar, na.rm=T, keep.var = F)))

sqrt(coef(svyby(~sstesto, ~die_02, des.2, svyvar, na.rm=T, keep.var = F)))

sqrt(coef(svyby(~d.age, ~die_02, des.2, svyvar, na.rm=T, keep.var = F)))

sqrt(coef(svyby(~bmi, ~die_02, des.2, svyvar, na.rm=T, keep.var = F)))

sqrt(coef(svyby(~educ, ~die_02, des.2, svyvar, na.rm=T, keep.var = F)))

sqrt(coef(svyby(~sstesto, ~die_03, des.2, svyvar, na.rm=T, keep.var = F)))

sqrt(coef(svyby(~d.age, ~die_03, des.2, svyvar, na.rm=T, keep.var = F)))

sqrt(coef(svyby(~bmi, ~die_03, des.2, svyvar, na.rm=T, keep.var = F)))

sqrt(coef(svyby(~educ, ~die_03, des.2, svyvar, na.rm=T, keep.var = F)))

sqrt(coef(svyby(~sstesto, ~die_04, des.2, svyvar, na.rm=T, keep.var = F)))

sqrt(coef(svyby(~d.age, ~die_04, des.2, svyvar, na.rm=T, keep.var = F)))

sqrt(coef(svyby(~bmi, ~die_04, des.2, svyvar, na.rm=T, keep.var = F)))

sqrt(coef(svyby(~educ, ~die_04, des.2, svyvar, na.rm=T, keep.var = F)))

sqrt(coef(svyby(~sstesto, ~die_05, des.2, svyvar, na.rm=T, keep.var = F)))

sqrt(coef(svyby(~d.age, ~die_05, des.2, svyvar, na.rm=T, keep.var = F)))

sqrt(coef(svyby(~bmi, ~die_05, des.2, svyvar, na.rm=T, keep.var = F)))

sqrt(coef(svyby(~educ, ~die_05, des.2, svyvar, na.rm=T, keep.var = F)))

sqrt(coef(svyby(~sstesto, ~die_06, des.2, svyvar, na.rm=T, keep.var = F)))

sqrt(coef(svyby(~d.age, ~die_06, des.2, svyvar, na.rm=T, keep.var = F)))

sqrt(coef(svyby(~bmi, ~die_06, des.2, svyvar, na.rm=T, keep.var = F)))

sqrt(coef(svyby(~educ, ~die_06, des.2, svyvar, na.rm=T, keep.var = F)))

sqrt(coef(svyby(~sstesto, ~die_07, des.2, svyvar, na.rm=T, keep.var = F)))

sqrt(coef(svyby(~d.age, ~die_07, des.2, svyvar, na.rm=T, keep.var = F)))

sqrt(coef(svyby(~bmi, ~die_07, des.2, svyvar, na.rm=T, keep.var = F)))

sqrt(coef(svyby(~educ, ~die_07, des.2, svyvar, na.rm=T, keep.var = F)))

sqrt(coef(svyby(~sstesto, ~die_08, des.2, svyvar, na.rm=T, keep.var = F)))

sqrt(coef(svyby(~d.age, ~die_08, des.2, svyvar, na.rm=T, keep.var = F)))

sqrt(coef(svyby(~bmi, ~die_08, des.2, svyvar, na.rm=T, keep.var = F)))

sqrt(coef(svyby(~educ, ~die_08, des.2, svyvar, na.rm=T, keep.var = F)))

sqrt(coef(svyby(~sstesto, ~die_09, des.2, svyvar, na.rm=T, keep.var = F)))

sqrt(coef(svyby(~d.age, ~die_09, des.2, svyvar, na.rm=T, keep.var = F)))

sqrt(coef(svyby(~bmi, ~die_09, des.2, svyvar, na.rm=T, keep.var = F)))

sqrt(coef(svyby(~educ, ~die_09, des.2, svyvar, na.rm=T, keep.var = F)))

sqrt(coef(svyby(~sstesto, ~die_10, des.2, svyvar, na.rm=T, keep.var = F)))

sqrt(coef(svyby(~d.age, ~die_10, des.2, svyvar, na.rm=T, keep.var = F)))

sqrt(coef(svyby(~bmi, ~die_10, des.2, svyvar, na.rm=T, keep.var = F)))

sqrt(coef(svyby(~educ, ~die_10, des.2, svyvar, na.rm=T, keep.var = F)))

svyttest(sstesto~die_01, des.2)

svyttest(sstesto~die_02, des.2)

svyttest(sstesto~die_03, des.2)

svyttest(sstesto~die_04, des.2)

svyttest(sstesto~die_05, des.2)

svyttest(sstesto~die_06, des.2)

svyttest(sstesto~die_07, des.2)

svyttest(sstesto~die_08, des.2)

svyttest(sstesto~die_09, des.2)

svyttest(sstesto~die_10, des.2)

svyttest(d.age~die_01, des.2)

svyttest(d.age~die_02, des.2)

svyttest(d.age~die_03, des.2)

svyttest(d.age~die_04, des.2)

svyttest(d.age~die_05, des.2)

svyttest(d.age~die_06, des.2)

svyttest(d.age~die_07, des.2)

svyttest(d.age~die_08, des.2)

svyttest(d.age~die_09, des.2)

svyttest(d.age~die_10, des.2)

svyttest(white~die_01, des.2)

svyttest(white~die_02, des.2)

svyttest(white~die_03, des.2)

svyttest(white~die_04, des.2)

svyttest(white~die_05, des.2)

svyttest(white~die_06, des.2)

svyttest(white~die_07, des.2)

svyttest(white~die_08, des.2)

svyttest(white~die_09, des.2)

svyttest(white~die_10, des.2)

svyttest(black~die_01, des.2)

svyttest(black~die_02, des.2)

svyttest(black~die_03, des.2)

svyttest(black~die_04, des.2)

svyttest(black~die_05, des.2)

svyttest(black~die_06, des.2)

svyttest(black~die_07, des.2)

svyttest(black~die_08, des.2)

svyttest(black~die_09, des.2)

svyttest(black~die_10, des.2)

svyttest(other~die_01, des.2)

svyttest(other~die_02, des.2)

svyttest(other~die_03, des.2)

svyttest(other~die_04, des.2)

svyttest(other~die_05, des.2)

svyttest(other~die_06, des.2)

svyttest(other~die_07, des.2)

svyttest(other~die_08, des.2)

svyttest(other~die_09, des.2)

svyttest(other~die_10, des.2)

svyttest(hisp~die_01, des.2)

svyttest(hisp~die_02, des.2)

svyttest(hisp~die_03, des.2)

svyttest(hisp~die_04, des.2)

svyttest(hisp~die_05, des.2)

svyttest(hisp~die_06, des.2)

svyttest(hisp~die_07, des.2)

svyttest(hisp~die_08, des.2)

svyttest(hisp~die_09, des.2)

svyttest(hisp~die_10, des.2)

svyttest(educ~die_01, des.2)

svyttest(educ~die_02, des.2)

svyttest(educ~die_03, des.2)

svyttest(educ~die_04, des.2)

svyttest(educ~die_05, des.2)

svyttest(educ~die_06, des.2)

svyttest(educ~die_07, des.2)

svyttest(educ~die_08, des.2)

svyttest(educ~die_09, des.2)

svyttest(educ~die_10, des.2)

svyttest(bmi~die_10, des.2)

svyttest(bmi~die_09, des.2)

svyttest(bmi~die_08, des.2)

svyttest(bmi~die_07, des.2)

svyttest(bmi~die_06, des.2)

svyttest(bmi~die_05, des.2)

svyttest(bmi~die_04, des.2)

svyttest(bmi~die_03, des.2)

svyttest(bmi~die_02, des.2)

svyttest(bmi~die_01, des.2)

#START HERE!!!!!!

#binomial model

#heart disease

svglm1b<-svyglm(I(die_01)~bs(start)*(sstesto)+black+other+hisp+educ+bmi, des.m2, family=binomial(link="cloglog"))

summary(svglm1b)

round(cbind(exp(confint(svglm1b))[,1], exp(coef(svglm1b)), exp(confint(svglm1b))[,2]), 2)

#cancer

svglm2b<-svyglm(I(die_02)~bs(start)*(sstesto)+black+other+hisp+educ+bmi, des.m2, family=binomial(link="cloglog"))

summary(svglm2b)

round(cbind(exp(confint(svglm2b))[,1], exp(coef(svglm2b)), exp(confint(svglm2b))[,2]), 3)

#chronic lower resp

svglm3b<-svyglm(I(die_03)~bs(start)*(sstesto)+black+other+hisp+educ+bmi, des.m2, family=binomial(link="cloglog"))

summary(svglm3b)

round(cbind(exp(confint(svglm3b))[,1], exp(coef(svglm3b)), exp(confint(svglm3b))[,2]), 2)

#accidents

svglm4b<-svyglm(I(die_04)~bs(start)*(sstesto)+black+other+hisp+educ+bmi, des.m2, family=binomial(link="cloglog"))

summary(svglm4b)

round(cbind(exp(confint(svglm4b))[,1], exp(coef(svglm4b)), exp(confint(svglm4b))[,2]), 2)

#cerebrovascular

svglm5b<-svyglm(I(die_05)~bs(start)*(sstesto)+black+other+hisp+educ+bmi, des.m2, family=binomial(link="cloglog"))

summary(svglm5b)

round(cbind(exp(confint(svglm5b))[,1], exp(coef(svglm5b)), exp(confint(svglm5b))[,2]), 2)

#alzheimer's

svglm6b<-svyglm(I(die_06)~bs(start)*(sstesto)+black+other+hisp+educ+bmi, des.m2, family=binomial(link="cloglog"))

summary(svglm6b)

round(cbind(exp(confint(svglm6b))[,1], exp(coef(svglm6b)), exp(confint(svglm6b))[,2]), 2)

#diabetes

svglm7b<-svyglm(I(die_07)~bs(start)*(sstesto)+black+other+hisp+educ+bmi, des.m2, family=binomial(link="cloglog"))

summary(svglm7b)

round(cbind(exp(confint(svglm7b))[,1], exp(coef(svglm7b)), exp(confint(svglm7b))[,2]), 2)

#flu and pneumonia

svglm8b<-svyglm(I(die_08)~bs(start)*(sstesto)+black+other+hisp+educ+bmi, des.m2, family=binomial(link="cloglog"))

summary(svglm8b)

round(cbind(exp(confint(svglm8b))[,1], exp(coef(svglm8b)), exp(confint(svglm8b))[,2]), 2)

#kidney disease

svglm9b<-svyglm(I(die_09)~bs(start)*(sstesto)+black+other+hisp+educ+bmi, des.m2, family=binomial(link="cloglog"))

summary(svglm9b)

round(cbind(exp(confint(svglm9b))[,1], exp(coef(svglm9b)), exp(confint(svglm9b))[,2]), 2)

#all cause

svglmallb<-svyglm(I(die_all)~bs(start)*(sstesto)+black+other+hisp+educ+bmi, des.m2, family=binomial(link="cloglog"))

summary(svglmallb)

round(cbind(exp(confint(svglmallb))[,1], exp(coef(svglmallb)), exp(confint(svglmallb))[,2]), 2)

dat2<-expand.grid(start=seq(0,100,10),sstesto=quantile(nh_all_merge2$sstesto, na.rm=T)[2:4], black=mean(nh_all_merge2$black), other=mean(nh_all_merge2$other),hisp=mean(nh_all_merge2$hisp), educ=mean(nh_all_merge2$educ, na.rm= T),bmi=mean(nh_all_merge2$bmi, na.rm= T))

dat2$fitted<-as.numeric(predict(svglm1b, type = "response", newdata=dat2)) #heart

dat2$fitted2<-as.numeric(predict(svglm2b, type = "response", newdata=dat2)) #cancer

dat2$fitted3<-as.numeric(predict(svglm3b, type = "response", newdata=dat2)) #lower respiratory

dat2$fitted4<-as.numeric(predict(svglm4b, type = "response", newdata=dat2)) #accidents

dat2$fitted5<-as.numeric(predict(svglm5b, type = "response", newdata=dat2)) #cerebrovascular

dat2$fitted6<-as.numeric(predict(svglm6b, type = "response", newdata=dat2)) #alzheimer's

dat2$fitted7<-as.numeric(predict(svglm7b, type = "response", newdata=dat2)) #diabetes

dat2$fitted8<-as.numeric(predict(svglm8b, type = "response", newdata=dat2)) #influenza and pneumonia

dat2$fitted9<-as.numeric(predict(svglm9b, type = "response", newdata=dat2)) #nephritis

dat2$fittedall<-as.numeric(predict(svglmallb, type = "response", newdata=dat2)) #allcause

dat2$age<-as.numeric(as.character(dat2$start))

head(dat2)

png("C:/Users/Xaust/Box/NHANES/Updated Mortality Files 2015/Figure1.png", width=2400, height=1600, res = 150)

par(mar = c(5.1,4.1,4.1,2.1)+0.5)

#all cause

plot(dat2$age[1:11], dat2$fittedall[1:11], yaxt = "n", type="b", ylab="Hazard",lwd=2.5, xlab="Age", col="red", cex.lab = 2, cex.axis = 1.75, cex.main = 2, main =c("All Cause Mortality"), ylim=c(0, 0.8))

points(dat2$age[23:33], dat2$fittedall[23:33], type="b", col="blue", lwd=2.5)

legend("topleft", legend=c("Low Testosterone", "High Testosterone"), col=c("red", "blue"),cex = 1.75, lwd=1.75,lty=1)

axis(2, at = c(0, 0.2, 0.4, 0.6, 0.8), cex.axis = 1.75)

dev.off()

png("C:/Users/Xaust/Box/NHANES/Updated Mortality Files 2015/Figure2.png", width=1600, height=2400, res = 150)

par(mfrow=c(3,1), mar = c(5,8,5,5) + 0.1)

#heart

plot(dat2$age[1:11], dat2$fitted[1:11], yaxt = "n", type="b", ylab="Hazard",lwd=2.5, xlab="Age", col="red", cex.lab = 2,cex.axis = 1.75, cex.main = 2, main =c("Diseases of the Heart"), ylim=c(0, .4))

points(dat2$age[23:33], dat2$fitted[23:33], type="b",lwd=2.5, col="blue")

legend("topleft", legend=c("Low Testosterone", "High Testosterone"), col=c("red", "blue"), cex = 1.75,lwd=2,lty=1)

axis(2, at = c(0, 0.1, 0.2, 0.3, 0.4), cex.axis = 1.75)

#Cerebrovascular disease

plot(dat2$age[1:11], dat2$fitted5[1:11], yaxt = "n", type="b", ylab="Hazard",lwd=2.5, xlab="Age", col="red", cex.lab = 2, cex.axis = 1.75, cex.main = 2, main =c("Cerebrovascular Disease"), ylim=c(0, .16))

points(dat2$age[23:33], dat2$fitted5[23:33], type="b", col="blue", lwd=2.5)

legend("topleft", legend=c("Low Testosterone", "High Testosterone"), col=c("red", "blue"),cex = 1.75, lwd=1.75,lty=1)

axis(2, at = c(0, 0.04, 0.08, 0.12, 0.16), cex.axis = 1.75)

#cancer

plot(dat2$age[1:11], dat2$fitted2[1:11], yaxt = "n", type="b", ylab="Hazard",lwd=2.5, xlab="Age", col="red", cex.lab = 2, cex.axis = 1.75, cex.main = 2, main =c("Malignant Neoplasms"), ylim=c(0, .72))

points(dat2$age[23:33], dat2$fitted2[23:33], type="b", col="blue", lwd=2.5)

legend("topleft", legend=c("Low Testosterone", "High Testosterone"), col=c("red", "blue"),cex = 1.75, lwd=1.75,lty=1)

axis(2, at = c(0, 0.18, 0.36, 0.54, 0.72), cex.axis = 1.75)

dev.off()

png("C:/Users/Xaust/Box/NHANES/Updated Mortality Files 2015/Figure3.png", width=1600, height=2400, res = 150)

par(mfrow=c(3,1), mar = c(5,8,5,5) + 0.1)

#influenza

plot(dat2$age[1:11], dat2$fitted8[1:11], yaxt = "n", type="b", ylab="Hazard",lwd=2.5, xlab="Age", col="red", cex.lab = 2, cex.axis = 1.75, cex.main = 2, main =c("Influenza and Pneumonia"), ylim=c(0, .12))

points(dat2$age[23:33], dat2$fitted8[23:33], type="b", col="blue", lwd=2.5)

legend("topleft", legend=c("Low Testosterone", "High Testosterone"), col=c("red", "blue"),cex = 1.75, lwd=1.75,lty=1)

axis(2, at = c(0, 0.03, 0.06, 0.09, 0.12), cex.axis = 1.75)

#chronic respiratory

plot(dat2$age[1:11], dat2$fitted3[1:11], yaxt = "n", type="b", ylab="Hazard",lwd=2.5, xlab="Age", col="red", cex.lab = 2, cex.axis = 1.75, cex.main = 2, main =c("Chronic Lower Respiratory Diseases"), ylim=c(0, .12))

points(dat2$age[23:33], dat2$fitted3[23:33], type="b", col="blue", lwd=2.5)

legend("topleft", legend=c("Low Testosterone", "High Testosterone"), col=c("red", "blue"),cex = 1.75, lwd=1.75,lty=1)

axis(2, at = c(0, 0.03, 0.06, 0.09, 0.12), cex.axis = 1.75)

#alzheimers

plot(dat2$age[1:11], dat2$fitted6[1:11], yaxt = "n", type="b", ylab="Hazard",lwd=2.5, xlab="Age", col="red", cex.lab = 2, cex.axis = 1.75, cex.main = 2, main =c("Alzheimer's Disease"), ylim=c(0, .032))

points(dat2$age[23:33], dat2$fitted6[23:33], type="b", col="blue", lwd=2.5)

legend("topleft", legend=c("Low Testosterone", "High Testosterone"), col=c("red", "blue"),cex = 1.75, lwd=1.75,lty=1)

axis(2, at = c(0, 0.008, 0.016, 0.024, 0.032), cex.axis = 1.75)

dev.off()

png("C:/Users/Xaust/Box/NHANES/Updated Mortality Files 2015/FigureSI.png", width=1600, height=2400, res = 150)

par(mfrow=c(3,1), mar = c(5,8,5,5) + 0.1)

#accidents

plot(dat2$age[1:11], dat2$fitted4[1:11], yaxt = "n", type="b", ylab="Hazard",lwd=2.5, xlab="Age", col="red", cex.lab = 2, cex.axis = 1.75, cex.main = 2, main =c("Accidents and Injuries"), ylim=c(0, .12))

points(dat2$age[23:33], dat2$fitted4[23:33], type="b", col="blue", lwd=2.5)

legend("topleft", legend=c("Low Testosterone", "High Testosterone"), col=c("red", "blue"),cex = 1.75, lwd=1.75,lty=1)

axis(2, at = c(0, 0.03, 0.06, 0.09, 0.12), cex.axis = 1.75)

#diabetes

plot(dat2$age[1:11], dat2$fitted7[1:11], yaxt = "n", type="b", ylab="Hazard",lwd=2.5, xlab="Age", col="red", cex.lab = 2, cex.axis = 1.75, cex.main = 2, main =c("Diabetes Mellitus"), ylim=c(0, .004))

points(dat2$age[23:33], dat2$fitted7[23:33], type="b", col="blue", lwd=2.5)

legend("topleft", legend=c("Low Testosterone", "High Testosterone"), col=c("red", "blue"),cex = 1.75, lwd=1.75,lty=1)

axis(2, at = c(0, 0.001, 0.002, 0.003, 0.004), cex.axis = 1.75)

#Nephritis

plot(dat2$age[1:11], dat2$fitted9[1:11], yaxt = "n", type="b", ylab="Hazard",lwd=2.5, xlab="Age", col="red", cex.lab = 2, cex.axis = 1.75, cex.main = 2, main =c("Kidney Diseases"), ylim=c(0, .04))

points(dat2$age[23:33], dat2$fitted9[23:33], type="b", col="blue", lwd=2.5)

legend("topleft", legend=c("Low Testosterone", "High Testosterone"), col=c("red", "blue"),cex = 1.75, lwd=1.75,lty=1)

axis(2, at = c(0, 0.01, 0.02, 0.03, 0.04), cex.axis = 1.75)

dev.off()
