# Supplementary material for: Lower testosterone levels are associated with higher risk of death in men
Source: Evol Med Public Health. 2022 Dec 26;11(1):30–41. doi: 10.1093/emph/eoac044 (PMC9938530; doi:10.1093/emph/eoac044)
Supplement: eoac044_suppl_Supplementary_File_S2 [file eoac044_suppl_supplementary_file_s2.docx]

Supplemental information for:

**Lower testosterone levels are associated with higher risk of death in men**

Table S1. Descriptive statistics for sample

| **Variable** | **Alive or Censored**  **(*n* = 8,618)** | **Heart Disease  (n = 475)** | **Malignant Neoplasms (n = 395)** | **Chronic Lower Respiratory Diseases (n = 66)** | **Accidents/ Injuries (n = 52)** |
| --- | --- | --- | --- | --- | --- |
| Age (years) | 54.23 (16.51) | 72.17 (13.67)*** | 70.77 (13.40)*** | 73.57 (10.76)*** | 59.15 (18.78)* |
| Race/Ethnicity |  |  |  |  |  |
| NH White | 59.48% | 60.40% | 64.44% | 61.15% | 68.21% |
| NH Black | 9.21% | 7.95% | 5.59%* | 2.10%*** | 5.96% |
| NH Other | 7.31% | 3.08%*** | 3.09% | 6.36% | 1.73%*** |
| Hispanic/Latino | 24.00% | 28.57% | 26.88% | 30.39% | 24.10% |
| Education (1–3) | 2.45 (0.76) | 2.09 (0.85)*** | 2.21 (0.82)*** | 2.07 (0.82)** | 2.12 (0.84) |
| Body Mass Index (kg/m2) | 28.34 (5.99) | 28.96 (6.01) | 27.26 (5.02)* | 31.27 (8.65) | 28.32 (7.34) |
| C-Reactive Protein (mg/dL)^1^ | 0.31 (0.78) | 0.44 (0.79)** | 0.48 (0.80) | 0.47 (0.42)** | 0.84 (1.17)* |
| Testosterone (ng/mL) | 4.43 (2.01) | 4.00 (1.86)*** | 4.84 (2.37)* | 3.61 (2.15)* | 4.79 (2.21) |

*Note.* Asterisks denote significant difference from reference group. **p* < 0.05, ***p* < 0.01, ****p* < 0.001, †*p* < 0.06. ^1^Data for C-reactive protein were not available for the 2011-12, 2013-14, and 2015-2016 NHANES waves.

Table S2. Descriptive statistics for sample

| **Variable** | **Cerebrovascular Diseases (n = 86)** | **Alzheimer's Disease (n = 38)** | **Diabetes Mellitus (n = 49)** | **Influenza/ Pneumonia (n = 29)** | **Kidney Disease (n = 24)** |
| --- | --- | --- | --- | --- | --- |
| Age (years) | 75.63 (10.91)*** | 80.33 (10.02)*** | 67.51 (14.39)*** | 75.88 (10.39)*** | 72.55 (12.52)*** |
| Race/Ethnicity |  |  |  |  |  |
| NH White | 62.11% | 80.09%* | 69.95% | 52.12% | 61.93% |
| NH Black | 11.20% | 1.96%*** | 7.38% | 9.29% | 18.62% |
| NH Other | 4.14% | 0.00%*** | 0.00%*** | 7.54% | 5.06% |
| Hispanic/Latino | 22.55% | 17.95% | 22.67% | 31.05% | 14.39% |
| Education (1–3) | 2.18 (0.82) | 2.32 (0.81) | 2.14 (0.84) | 2.06 (0.82) | 2.01 (0.85)† |
| Body Mass Index (kg/m2) | 26.58 (4.32)* | 26.34 (3.97)** | 28.26 (6.67) | 28.83 (7.47) | 29.21 (4.67) |
| C-Reactive Protein (mg/dL)^1^ | 0.55 (0.91)*** | 0.22 (0.20)** | 0.28 (0.25) | 0.48 (0.88)* | 0.51 (0.43) |
| Testosterone (ng/mL) | 3.72 (1.65)** | 4.09 (1.74) | 4.02 (1.94) | 4.21 (1.51) | 4.45 (2.47) |

*Note.* Asterisks denote significant difference from reference group. **p* < 0.05, ***p* < 0.01, ****p* < 0.001, †*p* < 0.06. ^1^Data for C-reactive protein were not available for the 2011-12, 2013-14, and 2015-2016 NHANES waves.

Table S3. Results of discrete time hazard models for all-cause mortality, heart diseases, cardiovascular diseases, and malignant neoplasms for data including C-reactive Protein (NHANES III, 1999-2000, 2001-2002, 2003-2004)

|  | **All-Cause Mortality (n = 739)** | | | | **Heart Disease (n = 306)** | | | | **Cerebrovascular Disease (n = 56)** | | | | | **Malignant Neoplasms (n = 245)** | | | | |  |
| --- | --- | --- | --- | --- | --- | --- | --- | --- | --- | --- | --- | --- | --- | --- | --- | --- | --- | --- | --- |
| **Predictor** | ***β*** | ***SE*** | ***p*** | **Hazard** | ***β*** | ***SE*** | ***p*** | **Hazard** | | ***β*** | ***SE*** | ***p*** | **Hazard** | | ***β*** | ***SE*** | ***p*** | **Hazard** | |
|  |  |  |  | **Ratio (95% CIs)** |  |  |  | **Ratio (95% CIs)** | |  |  |  | **Ratio (95% CIs)** | |  |  |  | **Ratio (95% CIs)** | |
| **Age Spline-1** | 0.17 | 0.20 | 0.42 | 1.18 (0.80, 1.75) | 0.40 | 0.30 | 0.19 | 1.50 (0.83, 2.71) | | 0.40 | 0.49 | 0.42 | 1.49 (0.57, 3.90) | | 0.26 | 0.25 | 0.30 | 1.30 (0.79, 2.12) | |
| **Age Spline-2** | -0.79 | 0.44 | 0.08 | 0.45 (0.19, 1.08) | -1.25 | 0.66 | 0.06 | 0.29 (0.08, 1.04) | | -1.16 | 0.96 | 0.23 | 0.31 (0.05, 2.05) | | -0.94 | 0.49 | 0.06 | 0.39 (0.15, 1.03) | |
| **Age Spline-3** | 2.08 | 0.43 | <0.001** | 7.98 (3.41, 18.67) | 2.68 | 0.70 | <0.001** | 14.62 (3.74, 57.12) | | 1.68 | 1.02 | 0.11 | 5.39 (0.72, 40.15) | | 2.26 | 0.50 | <0.001** | 9.57 (3.58, 25.61) | |
| **Testosterone** | -0.13 | 0.03 | <0.001** | 0.88 (0.83, 0.93) | -0.27 | 0.04 | <0.001** | 0.76 (0.70, 0.82) | | -0.49 | 0.14 | 0.001** | 0.61 (0.46, 0.81) | | 0.00 | 0.03 | 0.98 | 1.00 (0.94, 1.07) | |
| **C-Reactive Protein** | 0.08 | 0.05 | 0.14 | 1.08 (0.98, 1.20) | 0.09 | 0.06 | 0.12 | 1.09 (0.98, 1.22) | | 0.14 | 0.04 | 0.003** | 1.15 (1.05, 1.25) | | 0.12 | 0.06 | 0.04* | 1.13 (1.01, 1.27) | |
| **African American** | -0.38 | 0.13 | 0.01** | 0.69 (0.53, 0.89) | -0.21 | 0.18 | 0.27 | 0.81 (0.57, 1.16) | | 0.31 | 0.25 | 0.22 | 1.36 (0.84, 2.22) | | -0.80 | 0.20 | <0.001** | 0.45 (0.30, 0.67) | |
| **Other Ethnicity** | -1.16 | 0.33 | 0.001** | 0.31 (0.16, 0.61) | -1.07 | 0.59 | 0.08 | 0.34 (0.11, 1.09) | | -15.70 | 0.36 | <0.001** | 0.00 (0.00, 0.00) | | -1.46 | 0.74 | 0.05† | 0.23 (0.05, 0.98) | |
| **Hispanic/Latino** | -0.42 | 0.12 | <0.001** | 0.66 (0.52, 0.83) | -0.42 | 0.22 | 0.06 | 0.66 (0.43, 1.01) | | -0.34 | 0.46 | 0.46 | 0.71 (0.29, 1.73) | | -0.44 | 0.19 | 0.03* | 0.64 (0.44, 0.94) | |
| **Education** | -0.44 | 0.06 | <0.001** | 0.65 (0.58, 0.72) | -0.57 | 0.11 | <0.001** | 0.57 (0.46, 0.70) | | -0.80 | 0.25 | 0.003** | 0.45 (0.27, 0.73) | | -0.28 | 0.10 | 0.01** | 0.76 (0.62, 0.91) | |
| **Body Mass Index** | -0.01 | 0.01 | 0.44 | 0.99 (0.96, 1.02) | 0.00 | 0.02 | 0.85 | 1.00 (0.96, 1.03) | | -0.01 | 0.03 | 0.67 | 0.99 (0.94, 1.04) | | -0.03 | 0.02 | 0.11 | 0.97 (0.93, 1.01) | |
| **Age 1*Testosterone** | -0.09 | 0.04 | 0.04* | 0.92 (0.85, 0.99) | -0.16 | 0.06 | 0.01* | 0.85 (0.76, 0.96) | | -0.19 | 0.11 | 0.08 | 0.82 (0.67, 1.02) | | -0.07 | 0.04 | 0.10 | 0.93 (0.86, 1.01) | |
| **Age 2*Testosterone** | 0.21 | 0.09 | 0.02* | 1.24 (1.05, 1.46) | 0.32 | 0.14 | 0.03* | 1.37 (1.05, 1.80) | | 0.33 | 0.19 | 0.10 | 1.39 (0.95, 2.03) | | 0.20 | 0.08 | 0.02* | 1.22 (1.04, 1.44) | |
| **Age 3*Testosterone** | -0.19 | 0.08 | 0.02* | 0.82 (0.70, 0.97) | -0.26 | 0.14 | 0.07 | 0.77 (0.59, 1.02) | | 0.14 | 0.21 | 0.49 | 1.15 (0.77, 1.73) | | -0.26 | 0.10 | 0.02* | 0.77 (0.63, 0.94) | |

*Note.* **p* < 0.05, ***p* < 0.01, ****p* < 0.001, †*p* < 0.06.

Table S4. Results of discrete time hazard models for influenza/pneumonia, chronic lower respiratory disease, and Alzheimer’s disease for data including C-reactive Protein (NHANES III, 1999-2000, 2001-2002, 2003-2004)

|  | **Influenza and Pneumonia (n = 22)** | | | | **Chronic Lower Respiratory Disease (n = 44)** | | | | **Alzheimer's Disease (n = 28)** | | | |
| --- | --- | --- | --- | --- | --- | --- | --- | --- | --- | --- | --- | --- |
| **Predictor** | ***β*** | ***SE*** | ***p*** | **Hazard** | ***β*** | ***SE*** | ***p*** | **Hazard** | ***β*** | ***SE*** | ***p*** | **Hazard** |
|  |  |  |  | **Ratio (95% CIs)** |  |  |  | **Ratio (95% CIs)** |  |  |  | **Ratio (95% CIs)** |
| **Age Spline-1** | 0.65 | 0.52 | 0.22 | 1.91 (0.70, 5.25) | 0.63 | 0.47 | 0.19 | 1.87 (0.74, 4.74) | 0.81 | 0.21 | <0.001** | 2.25 (1.50, 3.39) |
| **Age Spline-2** | -1.95 | 0.85 | 0.03* | 0.14 (0.03, 0.75) | -1.90 | 1.18 | 0.12 | 0.15 (0.01, 1.51) | -2.06 | 0.32 | <0.001** | 0.13 (0.07, 0.24) |
| **Age Spline-3** | 3.63 | 1.14 | 0.003** | 37.76 (4.05, 352.38) | 3.38 | 1.71 | 0.05† | 29.23 (1.03, 826.48) | 3.15 | 0.33 | <0.001** | 23.31 (12.25, 44.37) |
| **Testosterone** | -0.18 | 0.10 | 0.08 | 0.84 (0.69, 1.01) | -0.29 | 0.15 | 0.06† | 0.75 (0.56, 1.00) | -0.38 | 0.08 | <0.001** | 0.68 (0.59, 0.79) |
| **C-Reactive Protein** | 0.12 | 0.08 | 0.14 | 1.13 (0.96, 1.33) | 0.12 | 0.03 | <0.001** | 1.13 (1.06, 1.21) | -1.14 | 0.72 | 0.12 | 0.32 (0.08, 1.30) |
| **African American** | 0.07 | 0.51 | 0.89 | 1.07 (0.40, 2.92) | -0.81 | 0.48 | 0.1 | 0.44 (0.17, 1.14) | -2.02 | 0.21 | <0.001** | 0.13 (0.09, 0.20) |
| **Other Ethnicity** | -0.11 | 1.25 | 0.93 | 0.89 (0.08, 10.28) | -14.82 | 0.30 | <0.001** | 0.00 (0.00, 0.00) | -15.96 | 0.26 | <0.001** | 0.00 (0.00, 0.00) |
| **Hispanic/Latino** | -0.80 | 0.84 | 0.35 | 0.45 (0.09, 2.34) | 0.05 | 0.38 | 0.90 | 1.05 (0.50, 2.22) | -1.06 | 0.56 | 0.06 | 0.35 (0.12, 1.03) |
| **Education** | -0.61 | 0.22 | 0.01** | 0.54 (0.35, 0.84) | -0.78 | 0.22 | 0.001** | 0.46 (0.30, 0.71) | -0.38 | 0.25 | 0.14 | 0.69 (0.42, 1.12) |
| **Body Mass Index** | 0.05 | 0.03 | 0.08 | 1.05 (1.00, 1.11) | -0.02 | 0.06 | 0.73 | 0.98 (0.88, 1.09) | -0.06 | 0.04 | 0.18 | 0.94 (0.86, 1.03) |
| **Age 1*Testosterone** | -0.25 | 0.09 | 0.01* | 0.78 (0.65, 0.94) | -0.25 | 0.09 | 0.01** | 0.78 (0.65, 0.93) | -0.31 | 0.03 | <0.001** | 0.73 (0.69, 0.78) |
| **Age 2*Testosterone** | 0.57 | 0.17 | 0.002** | 1.76 (1.26, 2.46) | 0.53 | 0.21 | 0.02* | 1.70 (1.12, 2.57) | 0.54 | 0.06 | <0.001** | 1.71 (1.53, 1.92) |
| **Age 3*Testosterone** | -0.47 | 0.22 | 0.04* | 0.63 (0.41, 0.96) | -0.25 | 0.32 | 0.44 | 0.78 (0.42, 1.46) | -0.14 | 0.08 | 0.10 | 0.87 (0.73, 1.02) |

*Note.* **p* < 0.05, ***p* < 0.01, ****p* < 0.001, †*p* < 0.06.

Table S5. Results of discrete time hazard models for diabetes mellitus, kidney disease, and accidents/injuries for data including C-reactive Protein (NHANES III, 1999-2000, 2001-2002, 2003-2004)

|  | **Diabetes (n = 23)** | | | | **Kidney Disease (n = 15)** | | | | **Accidents/Injuries (n = 25)** | | | |
| --- | --- | --- | --- | --- | --- | --- | --- | --- | --- | --- | --- | --- |
| **Predictor** | ***β*** | ***SE*** | ***p*** | **Hazard** | ***β*** | ***SE*** | ***p*** | **Hazard** | ***β*** | ***SE*** | ***p*** | **Hazard** |
|  |  |  |  | **Ratio (95% CIs)** |  |  |  | **Ratio (95% CIs)** |  |  |  | **Ratio (95% CIs)** |
| **Age Spline-1** | -0.90 | 0.27 | 0.002** | 0.41 (0.24, 0.69) | 1.40 | 1.34 | 0.31 | 4.04 (0.29, 56.33) | 0.03 | 0.32 | 0.92 | 1.03 (0.56, 1.92) |
| **Age Spline-2** | 1.32 | 0.66 | 0.05† | 3.73 (1.03, 13.55) | -2.12 | 3.78 | 0.58 | 0.12 (0.00, 199.00) | 0.18 | 1.78 | 0.92 | 1.19 (0.04, 39.39) |
| **Age Spline-3** | 2.05 | 1.13 | 0.08 | 7.76 (0.85, 70.96) | 1.33 | 4.01 | 0.74 | 3.78 (0.00, 9797.46) | 3.17 | 2.88 | 0.28 | 23.93 (0.08, 6740.70) |
| **Testosterone** | 0.02 | 0.06 | 0.68 | 1.02 (0.91, 1.15) | 0.03 | 0.19 | 0.89 | 1.03 (0.71, 1.48) | -0.09 | 0.17 | 0.61 | 0.91 (0.65, 1.29) |
| **C-Reactive Protein** | -0.08 | 0.22 | 0.71 | 0.92 (0.59, 1.42) | 0.15 | 0.08 | 0.06 | 1.16 (1.00, 1.36) | 0.20 | 0.09 | 0.03* | 1.22 (1.02, 1.45) |
| **African American** | 0.30 | 0.56 | 0.59 | 1.35 (0.45, 4.07) | 0.33 | 0.49 | 0.50 | 1.39 (0.54, 3.60) | -1.07 | 0.22 | <0.001** | 0.34 (0.22, 0.53) |
| **Other Ethnicity** | -15.53 | 0.26 | <0.001** | 0.00 (0.00, 0.00) | 0.22 | 1.25 | 0.86 | 1.24 (0.11, 14.39) | -14.78 | 0.25 | <0.001** | 0.00 (0.00, 0.00) |
| **Hispanic/Latino** | -1.06 | 0.78 | 0.18 | 0.35 (0.07, 1.61) | -1.95 | 0.94 | 0.04* | 0.14 (0.02, 0.89) | -0.69 | 0.46 | 0.14 | 0.50 (0.20, 1.23) |
| **Education** | -0.53 | 0.31 | 0.10 | 0.59 (0.32, 1.09) | -0.73 | 0.26 | 0.01** | 0.48 (0.29, 0.80) | 0.05 | 0.29 | 0.85 | 1.06 (0.60, 1.85) |
| **Body Mass Index** | 0.03 | 0.06 | 0.67 | 1.03 (0.91, 1.15) | 0.07 | 0.04 | 0.07 | 1.07 (1.00, 1.15) | 0.05 | 0.02 | 0.05† | 1.05 (1.00, 1.09) |
| **Age 1*Testosterone** | 0.07 | 0.06 | 0.28 | 1.07 (0.95, 1.21) | -0.30 | 0.21 | 0.17 | 0.74 (0.49, 1.13) | 0.05 | 0.10 | 0.62 | 1.05 (0.86, 1.28) |
| **Age 2*Testosterone** | -0.08 | 0.14 | 0.58 | 0.93 (0.71, 1.21) | 0.41 | 0.65 | 0.53 | 1.50 (0.42, 5.32) | -0.26 | 0.42 | 0.54 | 0.77 (0.34, 1.75) |
| **Age 3*Testosterone** | -0.36 | 0.23 | 0.12 | 0.70 (0.44, 1.09) | 0.06 | 0.72 | 0.93 | 1.06 (0.26, 4.38) | -0.33 | 0.60 | 0.58 | 0.72 (0.22, 2.32) |

*Note.* **p* < 0.05, ***p* < 0.01, ****p* < 0.001, †*p* < 0.06.

**Figure S1.** Hazard ratios by testosterone level for accidents/injuries, diabetes mellitus, and kidney diseases.


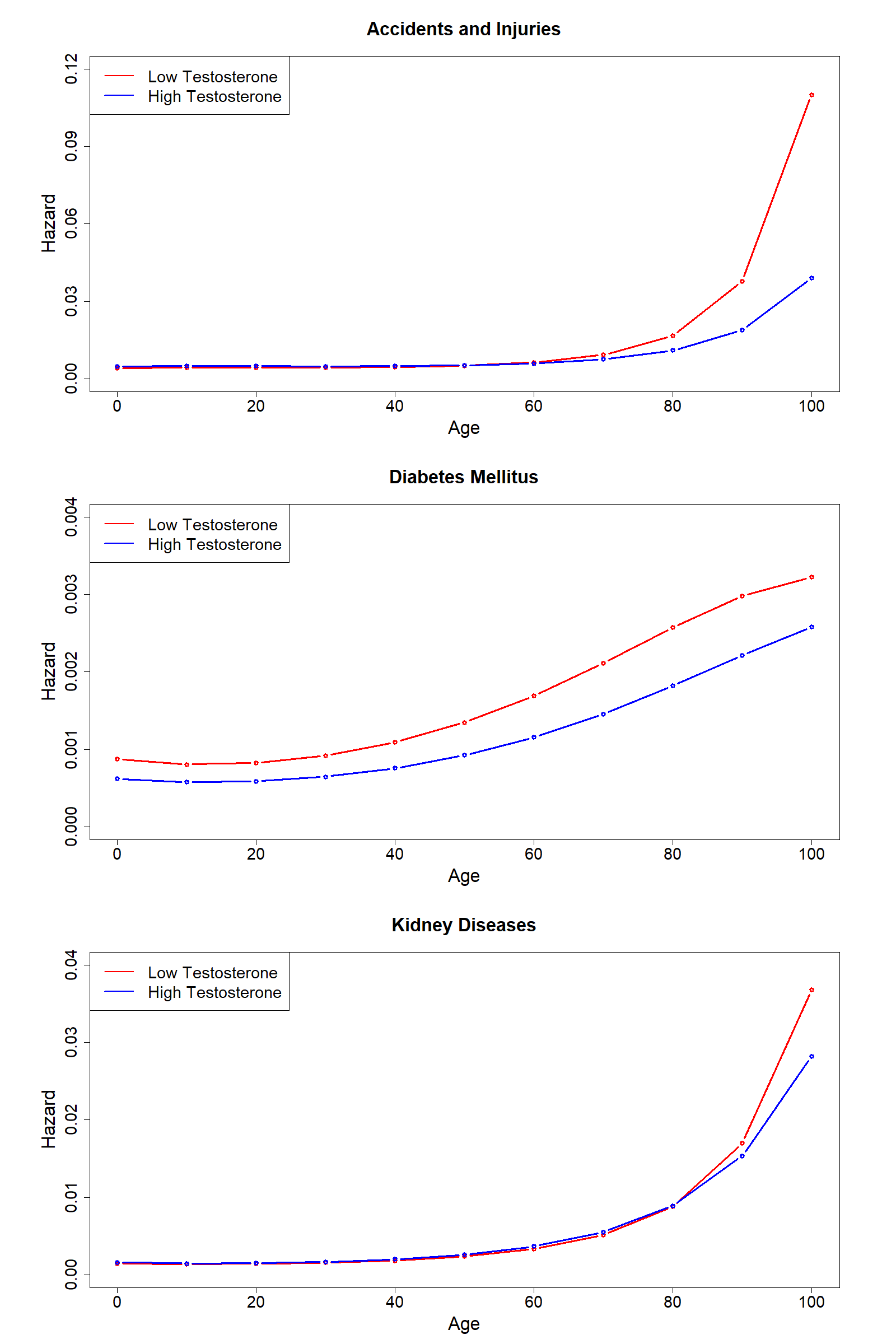


*Note.* Y axes were modified between categories to improve visibility of effects. High and low levels reflect quantiles at cutpoints of 25% and 75% of testosterone variable.
